# TRIM28 regulates the coagulation cascade inhibited by p72 of African swine fever virus

**DOI:** 10.1186/s13567-024-01407-6

**Published:** 2024-11-12

**Authors:** Xuejiao Zhu, Fang Li, Baochao Fan, Yongxiang Zhao, Junming Zhou, Dandan Wang, Renqiang Liu, Dongming Zhao, Huiying Fan, Bin Li

**Affiliations:** 1 Institute of Veterinary Medicine, Jiangsu Academy of Agricultural Sciences, Key Laboratory of Veterinary Biological Engineering and Technology, Ministry of Agriculture, Nanjing, 210014 Jiangsu Province China; 2 Jiangsu Coinnovation Center for Prevention and Control of Important Animal Infectious Diseases and Zoonoses, Yangzhou, 225009 China; 3 State Key Laboratory for Animal Disease Control and Prevention, National African Swine Fever Para-Reference Laboratory, Harbin Veterinary Research Institute, Chinese Academy of Agricultural Sciences, Harbin, China; 4 Jiangsu Key Laboratory for Food Quality and Safety-State Key Laboratory Cultivation Base of the Ministry of Science and Technology, Nanjing, China; 5 School of Food and Biological Engineering, Jiangsu University, Zhenjiang, 212013 China; 6 School of Life Sciences, Jiangsu University, Zhenjiang, 212013 China; 7 College of Veterinary Medicine, South China Agricultural University, Guangzhou, 510642 China; 8 GuoTai (Taizhou) Center of Technology Innovation for Veterinary Biology, Taizhou, 225300 China

**Keywords:** ASFV, coagulation cascade, P72, factor 10, TRIM28

## Abstract

In 2018, African swine fever virus (ASFV) emerged in China, causing extremely serious economic losses to the domestic pig industry. Infection with ASFV can cause disseminated coagulation, leading to the consumption of platelets and coagulation factors and severe bleeding. However, the mechanism of virus-induced coagulation has yet to be established. In our study, ASFV downregulated the coagulation process, as detected by D-dimer (D2D) and Factor X (F10) expression in pigs challenged with ASFV HLJ/18. In vitro, ASFV infection increased Factor IX (F9) and Factor XII (F12) expression while downregulating F10 expression in porcine alveolar macrophages (PAMs). African swine fever virus induced both intrinsic and extrinsic coagulation cascades. In addition, several encoded proteins affect the expression of the crucial coagulation protein F10, and among the encoded proteins, p72 inhibits the activity and expression of F10. Proteomic analysis also revealed that p72 is involved in the coagulation cascade. p72 can interact with F10, and its inhibitory functional domains include amino acids 423–432 and amino acids 443–452. Finally, we found that F10 and p72 interact with tripartite motif-containing protein 28 (TRIM28). TRIM28 knockdown resulted in a decrease in F10 expression. Importantly, TRIM28 contributes to the reduction in F10 protein expression regulated by p72. Our findings revealed an inhibitory effect of the viral protein p72 on the ASFV infection-induced coagulation cascade and revealed a role of TRIM28 in reducing F10 expression, revealing a molecular mechanism of ASFV-associated coagulation.

**Supplementary Information:**

The online version contains supplementary material available at 10.1186/s13567-024-01407-6.

## Introduction

African swine fever (ASF) is one of the most important exotic animal diseases in China. ASF is an acute, febrile, and highly contagious disease of swine caused by African swine fever virus (ASFV), with an incidence rate of acute infection as high as 100% [1]. The general symptoms of infected pigs include high fever, respiratory disorders, multiple organ bleeding, and acute infection-related mortality. In August 2018, China reported the first outbreak of African swine fever, after which the epidemic spread rapidly, causing considerable losses to the Chinese pig industry [2–4]. African swine fever virus is the only member of the family *Asfarviridae* and the only known DNA arbovirus. It consists of a nucleoid, a protein core shell, an inner lipid membrane, an icosahedral protein capsid, and an outer lipid membrane [5]. The ASFV genome is a double-stranded DNA molecule of 170 to 190 kbp encoding more than 151–167 open reading frames, depending on the virus strain. Most functions of the encoded proteins are unknown [6]. The pathogenesis of ASFV is extremely complex, and ASFV has genotype diversity and antigen variability; to date, no effective vaccine or antiviral strategy has been approved for the prevention and control of ASFV infection, but several improved vaccine candidates are available. Recent research demonstrated that a recombinant experimental vaccine candidate [7–11], ASFV-G-ΔI177L, generated by deleting the I177L gene from the genome of the highly virulent pandemic ASFV strain first identified in Georgia by the United States Department of Agriculture, is able to protect pigs against the virulent ASFV isolate currently circulating and producing disease in Vietnam with similar efficacy as that reported for the Georgia strain [12, 13]. African swine fever virus usually leads to coagulation, but the mechanism is unknown. Systemic haemorrhage of multiple tissues and organs is a characteristic symptom of pigs infected with ASFV. Infection with ASFV is characterized by extensive bleeding and severe changes in the coagulation system, prolonged bleeding time, blood clot-impaired contractility, and decreased platelet aggregation [14]. In pigs infected with ASFV, bleeding can appear in areas such as the ear, nose, axilla, abdomen, perineum, tail, and foot [15, 16]. Characteristic bleeding may occur in the stomach, liver, kidney, and lymph nodes accompanied by splenomegaly and pulmonary oedema, all of which are consistent with disseminated intravascular coagulation (DIC) [17, 18].

In a physiological state, the coagulation system is an important part of innate immunity [19, 20]. When the body is exposed to exogenous or endogenous damage, the coagulation cascade reaction is activated to stop bleeding and prevent the infection of pathogenic microorganisms and the possible inflammatory response [21, 22]. Disruption of coagulation system function can cause severe damage to blood vessels and corresponding tissues and organs. Haemostasis comprises three elements: haemostasis, coagulation, and fibrinolysis [23]. The coagulation cascade is widely acknowledged. Most factors in the coagulation system are serine proteases. Coagulation results from a series of linked coagulation protease–zymogen reactions, eventually leading to the formation of active thrombin (FIIa), which transforms soluble fibrin into insoluble fibrin [24–26].

The coagulation activation process involves intrinsic and extrinsic pathways, both of which are involved in coagulation aggregation at the activation point of Factor X (F10) [27]. The thrombin formed by these two pathways is eventually transformed into fibrin in filament and crisscross forms and is filled with many blood cells to form blood clots [28]. Disseminated intravascular coagulation is one of the most pivotal factors in organ bleeding, as it leads to the consumption of coagulation factors and platelets, after which severe bleeding occurs [29]. Caused by the excessive activation of coagulation reactions, DIC leads to microvascular fibrin thrombosis, severe microcirculation failure due to the consumption of a large number of clotting factors and platelets, and secondary fibrin dissolution resulting in extensive systemic bleeding. The clinical outcomes of DIC include multiple organ dysfunction syndrome and death. In DIC, the coagulation pathways cause a large amount of thrombin production in a short time, with most of the process activated by tissue factor (TF). In addition to TF and other coagulants, inflammatory factors produced by different disease-related products can also affect the coagulation system and cause DIC. A procoagulant state can be defined by a series of alterations in the blood accompanied by increased levels of clotting factors (e.g., Factor VIII (F8) and Factor XI (F11)), soluble tissue factor and von Willebrand factor and decreasing levels of the natural anticoagulants protein C, protein S, antithrombin, or tissue factor pathway inhibitor. Furthermore, the levels of markers of thrombin generation (prothrombin fragment 1 t 2 and thrombin-antithrombin complexes), platelet activation, fibrin degradation and fibrinolysis (e.g., D-dimer (D2D) and plasmin-a2-antiplasmin complexes) are also increased. Elevated levels of multiple coagulation marker complexes have been detected in non-haemorrhagic [30–32] (e.g., influenza, human immunodeficiency virus, hepatitis C virus) and haemorrhagic [33–36] (e.g., Ebola and dengue-haemorrhagic viruses) viral infections, indicating that the coagulation system is activated.

Previous research has shown that ASFV infection also causes characteristic DIC events, such as coagulation factor and platelet consumption, leading to multiple types of tissue bleeding and organ dysfunction [17, 37, 38]. Early studies have shown that ASFV infection can decrease thrombin and platelet levels. The blood coagulation time was prolonged, leading to extensive bleeding, which became more severe with increased virulence [17, 37, 39]. In a recent study, ASFV OURT 88/3- and Benin-infected animals containing various coagulation cascade-related genes were differentially expressed in extracellular vesicles compared with those in the control group. There is a close relationship between ASFV infection and coagulation reactions [40]. Systemic multiple-organ haemorrhage is a characteristic symptom of ASFV infection in pigs [15]. Disseminated intravascular coagulation may be an important response to ASFV infection, but its specific molecular mechanism is still unclear. The liver is considered to be the main producer of most complement and coagulation components. Myeloid cells, such as monocytes and macrophages, are central innate immune cells that are not only responsible for eradicating invading pathogens but also crucial for initiating both inflammatory and coagulant responses [41–44]. The excessive activation of host innate immune and coagulation responses can lead to multiorgan failure and death [45, 46].

Tripartite motif-containing protein 28 (TRIM28) is a large multidomain protein that shares many structural features with three other TRIM proteins, TRIM24 (TIF1α), TRIM33 (TIF1γ), and TRIM66 (TIF1δ), which together constitute a Transcriptional Intermediary Factor 1 (TIF1) family [47, 48]. The TRIM28 protein contains four conserved structural domains, including a RING finger, two B-boxes, and a leucine zipper coiled-coil region (CC) at the amino (N) terminus [49]. TRIM28 has a plant homeodomain (PHD) finger and a bromodomain at its carboxyl (C) terminus. The PHD finger, as well as the RING domain, is a cysteine/histidine-rich structure that endows TRIM28 with intrinsic E3 ubiquitin ligase (RING domain) and SUMO E3 ligase (through the PHD) activity [50, 51].

Our study revealed that ASFV could reduce F10 and D2D expression in vivo and induce both intrinsic and extrinsic coagulation cascades in vitro. In addition, several encoded proteins affect the expression of the crucial coagulation protein F10, and among the encoded proteins, p72 is involved in the coagulation cascade by downregulating F10 expression and interacting with F10. We also revealed a special role for TRIM28 in mediating F10 degradation and revealed a novel mechanism of coagulation regulated by ASFV.

## Materials and methods

### Viruses and cell culture

The ASFV strain GZ201801 used in the in vitro study (GenBank: MT496893.1), which belongs to Genotype II, was obtained from South China Agricultural University [52]. The ASFV HLJ/18 used in the animal study is a genotype II strain (GenBank: MK333192), which was isolated and preserved by the State Key Laboratory for Animal Disease Control and Prevention, Harbin Veterinary Research Institute [18].

ASFV was propagated in porcine alveolar macrophage (PAM) cells. Porcine alveolar macrophages were collected from 20–30-day-old specific pathogen-free (SPF) pigs as previously described [53], and the cells were maintained in 10% foetal bovine serum (GIBCO, Invitrogen Corporation, CA, USA) RPMI 1640 medium (Thermo Scientific, USA) supplemented with 1% glutamine. Human embryonic kidney cells (293-T), Huh7 cells, porcine kidney cells (PK-15), 3D4 cells, and porcine iliac artery endothelial cells (American Culture Collection, Manassas, VA, USA) were grown in Dulbecco’s modified Eagle’s medium supplemented with 10% fetal bovine serum, 250 U/mL penicillin, and 250 μg/mL streptomycin (Thermo Fisher). The cells were maintained at 37 °C in a humidified atmosphere supplemented with 5% CO_2_.

### Plasmids

The different open reading frames of the ASFV strains GZ201801 and F10 (*Sus scrofa*) were synthesized by the Shanghai Genescript Company. The F10 promoter was constructed in the pGL-Basic 6 plasmid and sequenced correctly.

### Antibodies and reagents

FLAG rabbit polyclonal antibody (Cat No AF0036) was purchased from Abmart (Shanghai, China); anti-F10 primary antibody was purchased from Beyotime (Cat No AF6831) (Wuhan, China); and anti-HA (Cat No51064-2-AP), anti-TRIM28 (Cat No 15202-1AP), anti-F12 (Cat NO 12551-1-AP), anti-F9 (Cat No 21481-1-AP), anti-F7 (Cat No 23058-1-AP), anti-F2 (Cat No 66509-1-lg), and anti-F8 (Cat NO 66722-1-lg) primary antibodies were purchased from Proteintech (Wuhan, China). An anti-F11 antibody was purchased from Sino Biological (Cat No 10302-RP01). The pig coagulation F10 ELISA kit, pig coagulation factor II ELISA kit, and D-dimer ELISA kit were purchased from Cusabio Company (Wuhan, China). The inhibitor MG132 (Cat No. S2619) was purchased from Selleck (Shanghai, China).

### Quantitative PCR

The same amounts of cell and supernatant lysates for viral load detection were collected, and DNA was extracted. The data were analysed using the absolute quantitative polymerase chain reaction (qPCR) method. SYBR Green qPCR mix (Vazyme, China) was used according to the manufacturer’s recommendations. The reaction procedure was as follows: 95 °C for 10 s, followed by 40 cycles at 95 °C for 10 s, 55 °C for 30 s, and 72 °C for 30 s. Quantitative PCR was performed on an ABI Q6 (Applied Biosystems, Foster City, CA, USA). Porcine alveolar macrophages were collected for RNA extraction, and 200 ng of each RNA sample was reverse transcribed. The RNA reverse transcription products of PAMs were subjected to qPCR. Data analysis was conducted using the 2^−ΔΔCt^ comparative threshold method, and gene expression was normalized to the level of β-actin (Actb) mRNA. The results are presented as the fold change in relative expression compared with that in the mock group.

### Western blot

PAM cells or other proteins were collected in lysis buffer containing 1% protein inhibitor. A total of 20–30 μg of cell lysate protein from each sample was subjected to 10–12% sodium dodecyl sulphate‒polyacrylamide gel electrophoresis (SDS‒PAGE) and transferred onto 0.22-μm nitrocellulose membranes (Pall, Port Washington, NY, USA). The membranes were then incubated with 5% non-fat milk at room temperature for 2 h, washed three times with phosphate-buffered saline (PBS) containing 0.05% Tween 20, incubated with primary corresponding antibodies overnight at 4 °C, washed three times with PBS-T and incubated with a horseradish peroxidase-conjugated goat anti-mouse (or rabbit) IgG secondary antibody at room temperature for 1 h. Detection was performed using an enhanced chemiluminescence system (Thermo Fisher Scientific).

### siRNA

RNA interference was performed by transfecting siRNA fragments of coagulation factors F1–F12 and control siRNA. The siRNA sequences used are listed in Table 1. siRNA fragments (1 μg) were transfected into PAM cells using Rfect siRNA Transfection Reagent (BAIDAI, China). The efficacy of RNA interference was evaluated by Western blot analysis.

**Table 1:** **siRNAs used in the study**

siRNA	sequence
si-ssc-F3_001	TCAGATAAGCCCTAGACTA
si-ssc-F3_002	CTTCGAGTACAGGAAAGAA
si-ssc-F3_003	GCACTACAGATGTAATTGT
si-ssc-F7_001	TCTGGGTTTCCTACAACGA
si-ssc-F7_002	AACTGCGAAACCAACAAGA
si-ssc-F7_003	GAGCCCACAGTTGAATATC
si-ssc-F8_001	TACCCTGAAGAACATGGCT
si-ssc-F8_002	GAGAAGGAAGACGATAAAG
si-ssc-F8_003	ACCTGGTGAAAGACCTGAA
si-ssc-F9_001	CCCAATCCATGCTTAAACG
si-ssc-F9_002	TCATCTGACGACTTTATTC
si-ssc-F9_003	GCGGTTCCATCATTAATGA
si-ssc-F11_001	GCAATGCTCACACCAAATA
si-ssc-F11_002	CTACCATGACACTGAATTC
si-ssc-F11_003	GGATACAGAAGCCACAAGA
si-ssc-F12_001	TGAAAGACCACTGCAACAA
si-ssc-F12_002	CAATCCTTACTCCGACCCA
si-ssc-F12_003	GTATTACAAGTGCATCCAA
siTRIM28	TGGCTCTGTTCTCTGTCCT

### Animal experiments

The virulence of ASFV HLJ/18 was assessed in 8-week-old SPF pigs. Six pigs (numbered 1–6) were intramuscularly (IM) inoculated with 10^2.5^ HAD_50_ of ASFV HLJ/18. The development of ASF clinical symptoms (anorexia, depression, fever, purple skin discolouration, staggering gait, diarrhoea, and cough) and body temperature were recorded daily throughout the experiment. Blood samples were collected at 0, 3, 5, and 7 dpi to measure ASFV genomic DNA copy numbers; F10 and F10 activity; and Factor II (F2) and D2D expression by enzyme-linked immunosorbent assay (ELISA).

### Enzyme-linked immunosorbent assay

Blood samples from pigs challenged with ASFV HLJ/18 were collected at 0, 3, 5, and 7 dpi to measure F10 and F10 activity and F2 and D2D expression by ELISA, and ELISA was also performed within the enhanced biosafety level 3 (P3) group. All coagulation factor or related factor analyses were performed via commercial ELISA kits, and the procedures were conducted in strict accordance with the manufacturer’s instructions. The supernatants of Huh7 cells transfected with different plasmids were collected for F10 detection.

### In vitro study

PAM cells were isolated following a previously described method [54], and the cells were incubated in 24-well cell culture plates at 5 × 10^5^ cells per well and then infected with 0.1 MOI ASFV for 6, 12, 24, or 36 h. The infected cells and mock cells were collected at the indicated time points for western blot analysis and qPCR.

### Confocal microscopy

293-T cells were co-transfected with 1 μg of pcDNA3.1-F10-FLAG, pcDNA3.1-p72-HA, or mock plasmid in 24-well culture plates for 24 h. Alternatively, 293-T cells were transfected with p72-HA or F10-FLAG. At 24 h post-transfection, the cells were processed for confocal microscopy by fixation in 4% paraformaldehyde for 30 min. The cell membranes were permeabilized in PBS containing 0.2% Triton X-100 for 5 min. The fixed cells were incubated with anti-HA and anti-FLAG antibodies (1:500 diluted with PBS) or anti-TRIM28 at 37 °C for 1 h. After washing three times with PBS, Alexa Fluor 555-conjugated goat anti-rabbit IgG and Alexa Fluor 488-conjugated goat anti-mouse IgG mixed (at 1:400 dilution) were added to the target well and incubated for an additional 1 h. After washing three times with PBS, the cell nuclei were stained with DAPI for 5 min before viewing via laser-scanning confocal microscopy.

### Immunoprecipitation

P72 (151–450 aa) inhibits F10 expression. To determine whether the inhibitory effect depends on the interaction of p72 with F10, 293-T cells were transfected with 1 μg of pcDNA3.1-p72-HA (151–450 aa) or mock vector in 24-well culture plates per well. Alternatively, 293-T cells were transfected with 1 μg of p72 or F10-FLAG. At 24 h post-transfection, the cells were lysed in 100 μL of lysis buffer. The cell lysates were centrifuged (5000 × *g*, 5 min), 100 μL of the supernatant was collected as the input protein and preserved at -80 °C, and the remaining supernatants were incubated overnight at 4 °C with 1 μg of anti-HA antibodies (or anti-FLAG or anti-TRIM28) and then precoupled with 40 μL of A/G Plus agarose beads for 4 h at 4 °C. The immune complexes were precipitated, washed, and subjected to western blot analysis.

### Pull-down assay

The pig F10 protein is a commercial product (Elabscience, Shanghai, China), and the p72 protein (aa 321–451) and mock control protein (Rep protein derived from porcine circovirus type 2 with a His tag preserved by our laboratory) were secreted in a prokaryotic expression system and purified with His-tag purification resin. The same concentrations of p72 truncation or mock control were mixed with the F10 protein. The immune complexes were incubated overnight at 4 °C, precoupled with 40 μL of His-tag resin at 4 °C for another 4 h and subjected to western blot analysis.

### LC‒MS/MS analysis and data processing

pB646L (p72) of ASFV tagged with hemagglutinin (HA) at the C-terminus was synthesized into the plasmid pCDNA-3.1( +) by GenScript Corporation (Shanghai, China) and sequenced correctly. The 293-T cells were then separately transfected with pcDNA3.1( +)-p72-HA and pcDNA3.1( +) (1 μg per well, 12 μg in total) by Lipofectamine 3000 (Thermo Scientific, USA), and p72-expressing or empty vector-transfected cells were washed once in cold PBS and suspended in 1 mL of cold immunoprecipitation buffer (Beyotime, China) (50 mM Tris–HCl, pH 7.4; 150 mM NaCl; 1 mM EDTA) supplemented with 0.5% Nonidet P 40 Substitute (NP-40; Fluka Analytical) on ice with 1% protease inhibitor cocktail (Roche). The cells were then lysed for 30 min at 4 °C with constant rotation, and the lysates were cleared by centrifugation at 5000 × *g* for 5 min. The lysate was removed for western blot analysis (whole-cell lysate fraction). The remaining lysate was incubated with 1 μg of anti-HA antibody (Proteintech) overnight at 4 °C and then precoupled with 40 μL of A/G Plus agarose beads for 4 h at 4 °C according to the manufacturer’s instructions. The immune complexes were precipitated, washed, and subjected to SDS‒PAGE and western blot analysis. Peptides for mass spectrometry of triplicate samples from each group and for liquid chromatography‒mass spectrometry (LC‒MS/MS) were prepared by Shanghai Applied Protein Technology Company, and LC‒MS/MS was performed on a Q Exactive HF mass spectrometer (Thermo Scientific). The database of mock-interacting proteins was deleted for p72-interacting proteins to exclude possible contaminants or to specify the proteins that interact with p72. The raw data were processed via the tool Proteome Discoverer 1.4. GO enrichment analysis results were mapped to Gene Ontology (GO) terms in the Gene Ontology database, gene numbers were calculated for every term, and significantly enriched GO terms compared with those in the mock-transfected group were defined. The functional enrichment of the gene module and Kyoto Encyclopedia of Genes and Genomes (KEGG) pathway analyses of the interacting proteins were performed via the web-based tool DAVID.

### Effect of the MG132 inhibitor on p72-mediated F10 expression

MG132 is an inhibitor of proteasomes, and its working concentration is 100 nM. Huh7 cells were treated with MG132 (10, 50, or 100 nM) for 4 h prior to p72 truncation plasmid transfection. After transfection with p72 truncations for 24 h, the cell lysates were collected and subjected to western blot analysis.

### Effect of siTRIM28 on F10 expression

TRIM28 siRNA and NC were transfected into Huh7 cells at different doses (10, 50, and 75 nM). At 12 h post-transfection, the supernatant was removed, the medium was replaced with fresh medium, and the cells were then transfected with p72 (151–450 aa) or mock (1 μg). At 24 h post-transfection with p72 (151–450 aa), the cell lysates were collected and subjected to western blot analysis.

### Effect of siTRIM28 on F10 activity

TRIM28 and negative control siRNAs were transfected into Huh7 cells (50 nM). At 12 h post-transfection, the supernatant was removed, the medium was replaced with fresh medium, and the cells were co-transfected with the pGL-F10 promoter plasmid (1 μg) or pRL-TK (0.1 μg) with or without p72 (151–450 aa) (1 μg). At 24 h post-transfection, the cell lysates were collected and subjected to western blot and luciferase activity analyses. Luciferase activity analysis was performed following the instructions of the Promega Dual-luciferase® Reporter Assay System (Promega, USA).

### Statistical analysis

Statistical analysis was performed using GraphPad Prism version 5.0.2 (GraphPad Software, San Diego, CA, USA). Comparisons between groups were performed using the paired t tests and one-way analysis of variance. *p* < 0.05 was chosen to indicate statistical significance. All the data are expressed as the means ± standard deviations (SDs).

## Results

### Assessment of ASFV HLJ/18 virulence in pigs

**Figure 1:**
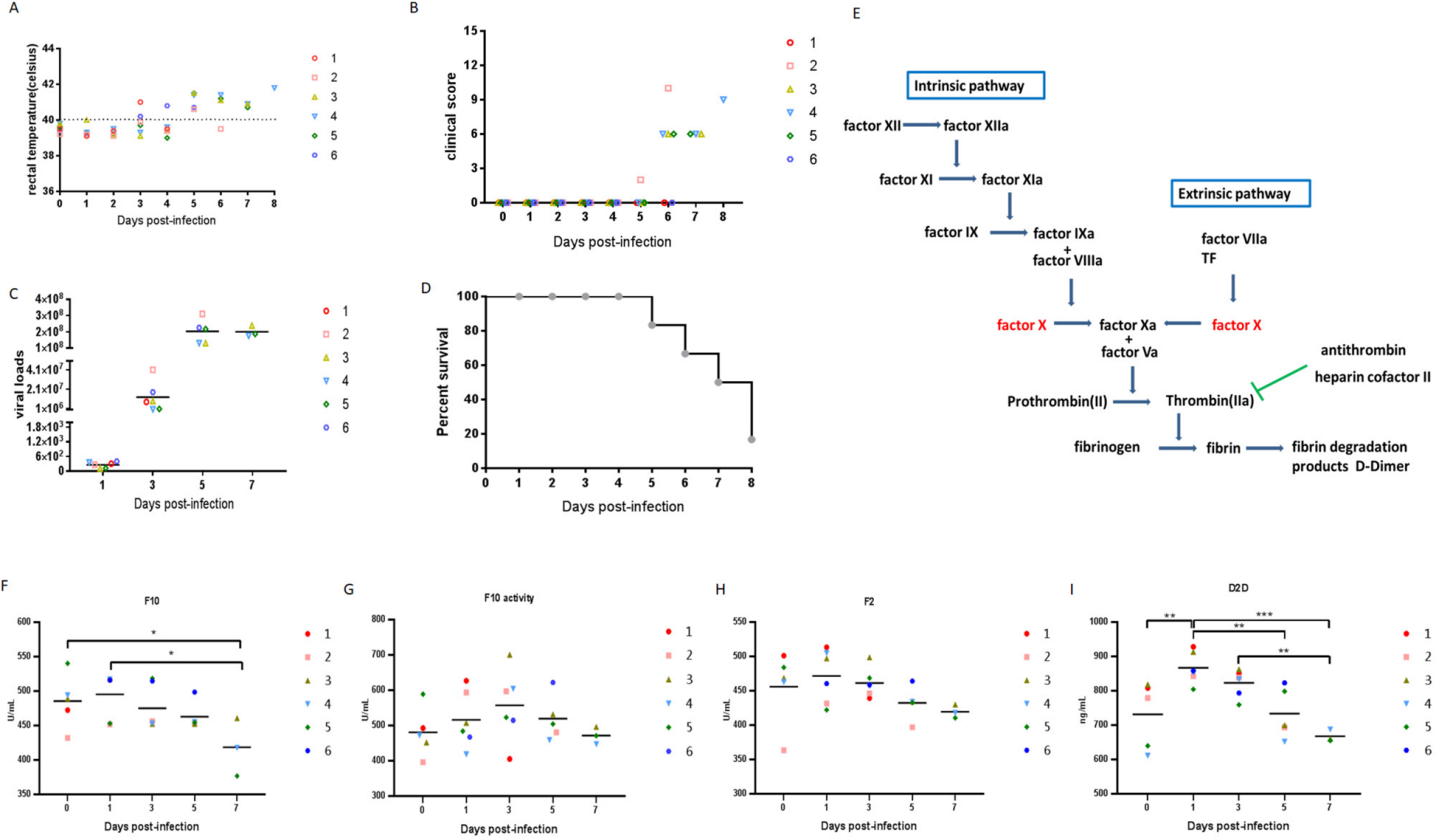
**ASFV infection regulated coagulation and related factors in vivo**. **A** Evolution of rectal temperature, **B** clinical score in animals (6 animals) IM infected with 10^2.5^ HAD_50_ of ASFV HLJ/18. **C** viremia, viremia values are expressed as copies/mL, sensitivity of virus detection: > 1000 copies/mL, and **D** mortality. Dpi: days post-infection. **E** Sketch of the coagulation activation process. **F** ASFV-positive serum samples from each pig at the indicated time points were collected for detection of F10 and **G** F10 activity. **H** ASFV-positive serum samples from each pig at the indicated time points were collected for detection of F2 and **I** D2D by ELISA. Statistical data were analysed by *t test* analysis of variance (*p < 0.05, **p < 0.01, and ***p < 0.001). All the data are expressed as the mean ± SD.

To evaluate the impact of ASFV on coagulation pathways, six 30–40 kg SPF pigs were inoculated IM with 10^2.5^ HAD_50_ of lethal ASFV HLJ/18. Two inoculated pigs (Nos. 1 and 6) presented a sudden fever (> 40 °C) at 3 dpi, and more pigs developed fever after 3 dpi, which was quickly followed by the development of full clinical disease at 5 dpi. The clinical score of each pig was evaluated (Figure 1A), and the clinical symptoms included depression (losing appetite and displaying lethargy), anorexia, diarrhoea, staggering gait, and purple skin discolouration (Figure 1B). Virus replication in animals experimentally infected with ASFV HLJ/18 was evaluated by quantifying viremia loads at different times post-infection. The viremia values were heterogeneous and followed the presentation of the clinical symptoms. As expected, viral loads in ASFV HLJ/18-infected animals started to increase at 1 dpi, peaked at 5 dpi and remained high at 7 dpi (approximately 2 × 10^8^ copies/mL), with only 3 pigs (Nos. 3, 4 and 5) living at that time (Figure 1C). Disease severity rapidly progressed to terminal disease; the No. 1 pig died at 5 dpi, the No. 6 pig died at 6 dpi, and only one (No. 4) of the six pigs that survived at 8 dpi with high body temperature developed clinical symptoms. All the animals were euthanized in extremis at 8 dpi (Figure 1D). Pathological changes were observed in euthanized animals, and the gross scores are shown in Table 2. As a result, the pathological scores were consistent with the clinical symptoms and viral loads.

**Table 2:** **Pathological scores of pigs infected with ASFV HLJ/18**

No.	Pathological scores								
	Lymph nodes	Heart	Spleen	Liver	Bladder	Kidney	Tonsil	Lung	Total
1	8	3	1	3	1	3	0	6	25
2	13	7	5	4	0	6	2	6	43
3	7	4	4	4	2	4	2	4	31
4	8	2	5	3	2	4	2	3	29
5	10	5	4	3	3	3	3	5	36
6	9	4	2	2	0	2	3	5	27

Gross scores of the tissues including lymph nodes (inguinal lymph node, submaxillary lymph node, mesenteric lymph node), the heart, spleen, liver, bladder, kidney, tonsil, and lung of pigs challenged with 10^2.5^ HAD_50_ of ASFV HLJ/18 (*n* = 6), were recorded and analysed.

### ASFV infection downregulates coagulation and related factors in vivo

The coagulation activation process consists of three main processes (Figure 1E), and multiple indices in each process are indicated. As previously described, D2D is a soluble fibrin degradation product of thrombi generated by the fibrinolytic system. The production or increase in D2D in the serum reflects the activation of the coagulation and fibrinolytic system. Compared with that at 0 dpi, F10 expression increased at 1 dpi but decreased at 3 dpi and reached its lowest level at 7 dpi when F10 expression was significantly lower than that at 0 and 1 dpi. For most of the pigs challenged with ASFV, F10 expression in vivo decreased in response to ASFV infection with increasing severity and disease development, except for one pig (No. 2), in which the initial F10 expression was quite low (Figure 1F). Surprisingly, a significant difference in F10 activity was not observed at the different time points; although F10 activity increased at 1 and 3 dpi, it decreased at 5 and 7 dpi. For each pig, F10 activity was very low before they were on the brink of their individual sacrifice except for No. 6, which died at 6 dpi, suggesting that F10 activity is preferentially downregulated by the severity of the disease in vivo (Figure 1G). We also found that the expression of coagulation factor F2 in the serum did not significantly change during the course of ASFV infection, although this trend was similar to that of F10 activity; that is, F2 expression increased slightly at 1 dpi and decreased at later time points (Figure 1H). For the detection of the degradation products of thrombi, the serum D2D expression level was significantly elevated after challenge with ASFV HLJ/18, gradually decreased at later days post-infection, and decreased to its lowest level at 7 dpi (Figure 1I). Collectively, these results suggest that ASFV HLJ/18 infection can induce coagulation and related factor expression in a short period of time while downregulating coagulation expression in pigs as the disease progresses and becomes more severe.

### ASFV infection regulates coagulation and related factors in vitro

**Figure 2:**
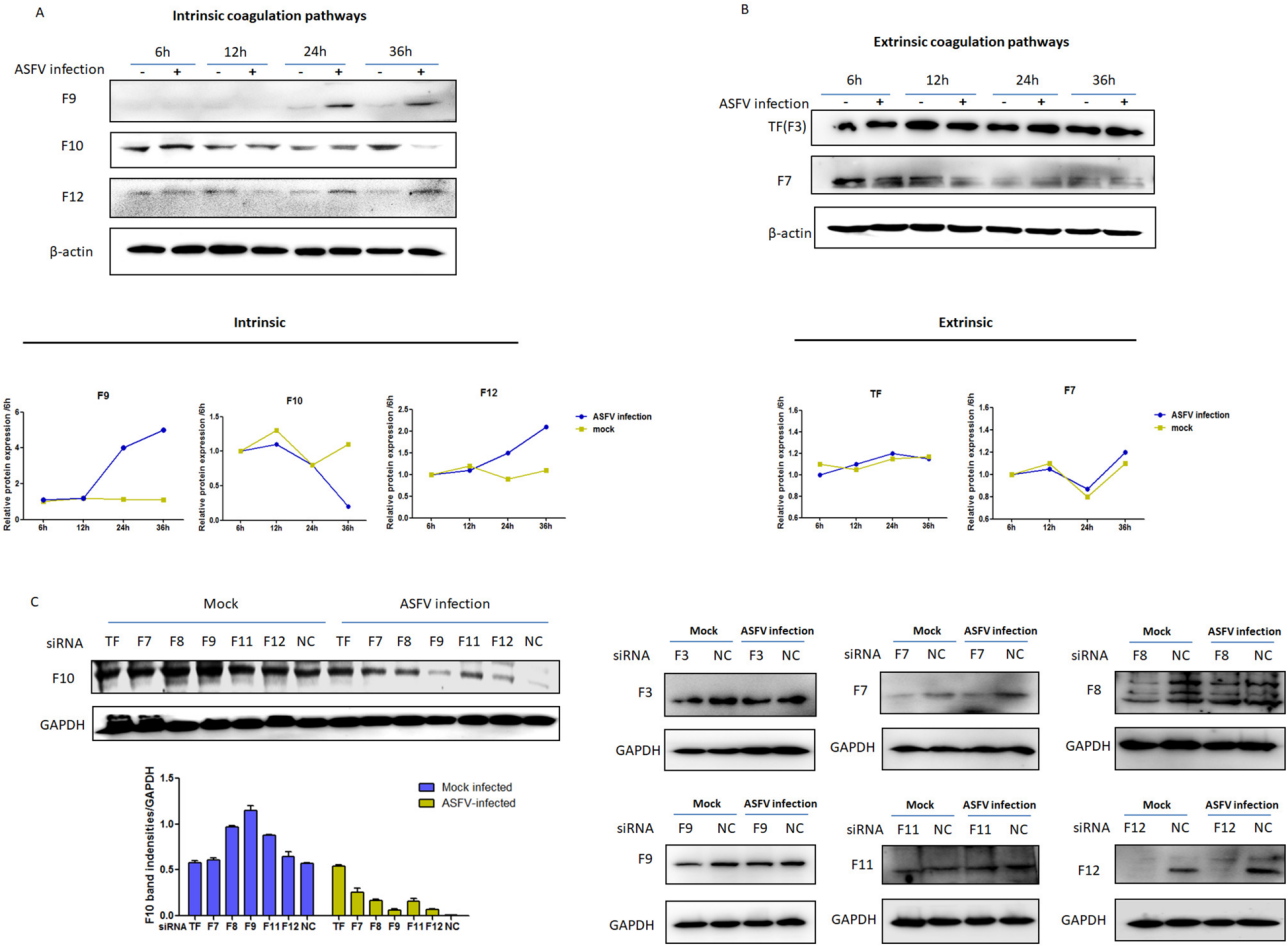
**ASFV infection regulated coagulation and related factors in vitro. PAM cells were infected with 0.1 MOI ASFV for 6, 12, 24, or 36 h**. **A** Intrinsic coagulation factors (F12 and F9), crucial coagulation factor F10, and **B** extrinsic coagulation factors (TF and F7) were detected by western blotting. The coagulation factor band intensities normalized to those of β-actin were scanned and analysed by ImageJ software and are shown in a line chart. **C** RNA interference was performed via the transfection of 1 μg of siRNA fragments encoding coagulation factors F1-F12 and control siRNA. siRNA-1 from each fragment was transfected into PAMs, which were then infected with 0.1 MOI ASFV or not, and at 36 hpi, the cells were lysed for western blot analysis of F10 expression and coagulation factor expression.

Porcine alveolar macrophages (PAMs) were collected and infected with ASFV for 6, 12, 24, or 36 h. The intrinsic coagulation factors were Factor XII (F12) and Factor IX (F9). The extrinsic coagulation factors are Factor III (F3), also known as tissue factor (TF), and Factor VII (F7). Factor X is a crucial coagulation factor and is the downstream component of both intrinsic and extrinsic coagulation factors. The protein level of F10 was significantly lower in ASFV-infected cells than in uninfected cells at 36 hpi (Figure 2A). In contrast, F9 and F12 protein levels were significantly greater in ASFV-infected cells than in uninfected cells at 24 and 36 hpi (Figure 2B). Thus, ASFV infection regulated coagulation and related factors in vitro.

### ASFV infection regulates intrinsic and extrinsic coagulation pathways

RNA interference was performed by transfecting siRNA fragments of coagulation factors F1–F12 and control siRNA. The best RNA interference efficiency of each coagulation factor was detected, as shown in Additional file 1. The optimal fragment was chosen to identify which coagulation pathway (intrinsic or extrinsic) affected ASFV infection. Previously, ASFV infection was shown to reduce F10 expression (Figure 2A), which is the key factor in the coagulation cascade. Surprisingly, F10 expression was slightly upregulated, which was expected to be reduced in F8-, F9-, and F11-siRNA-transfected cells, suggesting that there is a balance of coagulation between the procoagulant and (natural) anticoagulant. The optimal siRNA fragments of coagulation factors F1–F12 and the control siRNA were transfected into PAMs at 1 μg and infected with ASFV, and the results indicated that coagulation factor expression was knocked down by the siRNA fragments (Figure 2C). However, the expression of components of the intrinsic and extrinsic coagulation pathways was disrupted as ASFV infection occurred, with a decrease in F10 expression in coagulation factor siRNA-transfected cells but not in TF siRNA-transfected cells, suggesting that extrinsic coagulation factors play an important role in regulating F10 expression. In addition, compared with those in the siNC group upon ASFV infection, F10 expression recovered significantly in F7 (extrinsic pathway), F8, and F11 (intrinsic pathway) siRNA-transfected cells (Figure 2C), demonstrating that the downregulation of F10 expression induced by ASFV infection did not occur and that ASFV infection regulated both the intrinsic and extrinsic coagulation pathways.

### Several ASFV-encoded proteins regulate the coagulation process

**Figure 3:**
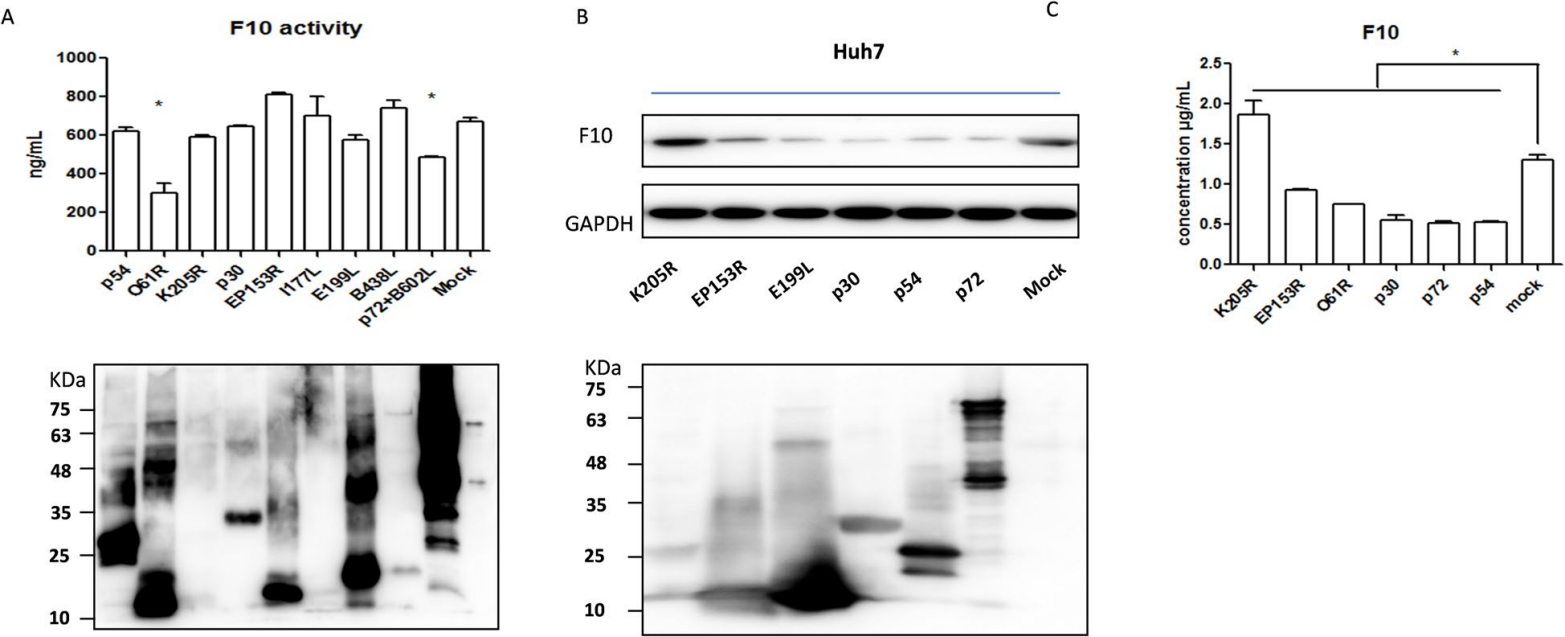
**ASFV-encoded proteins regulated F10 expression and activity**. **A** Several encoded proteins expressed in the baculovirus expression system were added to Huh7 cells at the same concentration for 24 h prior to supernatant and cell lysate collection. The mixtures were detected for F10 activity. **B** Huh7 cells were transfected with plasmids expressing ASFV-encoded proteins, including pK205R, pE199L, pEP153R, p30, p54, and p72, and then, the cells and supernatants were collected for detection of F10 expression by western blotting, **C** and secretory F10 expression by ELISA. For all figures, the experiments were repeated at least three times with similar results. The data are presented as the mean ± SD from one single experiment. Statistical significance was determined by Student’s *t* test (*p < 0.05).

As coagulation factors are produced by the liver and secreted into the blood circulation system, a proper cell line is needed to study the coagulation cascade. Thus, the Huh7 cell line derived from human liver tissue was selected for this study. ASFV encodes 151–167 open reading frames, and various viral proteins present challenges in the selection of target proteins that affect the coagulation process. In this study, we selected some viral proteins on the basis mainly of their function, structure and morphogenesis. For example, p72 + B602L (major capsid protein), p54 (inner envelope), and B438L (capsid) are related to the structure and morphogenesis of the virus [55], and the membrane proteins O61R (p12) and pE199L on the inner envelope are associated with virus entry [55, 56]. The structural protein p30 was membrane-localized and released into the culture medium shortly after infection. Phosphorylation, glycosylation, and membrane attachment sites are predicted by sequence analysis of p30 [57]. The unknown protein pI177L was recently reported to be related to the virulence of the virus [58]. K205R, located in viral factories, is expressed in the early stages of infection and can induce ER stress and activate autophagy [59]. EP153R is involved in the hemadsorption of infected cells [60]. Little is known about the coagulation process regulated by the ASFV viral protein, and we selected the proteins described above with various distributions and functions to identify potential regulatory alterations in F10 expression. Huh7 cells were grown in 24-well plates, and when the cells had grown to 80 ~ 90% fusion, baculovirus-expressing proteins (including p54, O61R, K205R, p30, EP153R, I177L, E199L, B438L, and p72 + B602L) at the same concentration (3 μg) were added to the cultured Huh7 cells for 24 h prior to supernatant and cell lysate collection. The mixtures containing the supernatant and cell freeze‒thaw products were centrifuged, and the F10 activity of the supernatants was detected at 24 h post-stimulation with an F10 activity detection kit according to the manufacturer’s instructions. The results showed that by directly stimulating Huh7 cells, the baculovirus expressed pO61R and p72 + B602L and could downregulate F10 activity despite the low expression of K205R, I177L, and B438L (Figure 3A). Interestingly, p72 and pO61R could affect F10 activity only by stimulating the cells. However, the possibility that other proteins are involved in F10 activity cannot be excluded because whether the proteins can enter cells or regulate F10 activity in the cell is still being determined. The reason that pO61R interferes with the activity of F10 is unknown. To determine whether p72 entered the cell, we added baculovirus-expressing p72 + B602L to PK-cell cultures for 0 min, 15, 30, 45 min, and 1 h. Then, at the indicated time points, the cells were fixed and probed with an anti-p72 antibody and stained green, and the cell borders were stained red with phalloidin. The results indicated that p72 could be detected in the PK-15 cytoplasm as early as 15 min after stimulation. After 1 h of stimulation, green fluorescence was observed, which was distributed mainly in the cytoplasm through the p72 protein but could not be detected widely in PK-15 cells at 1 h post-stimulation (Additional file 2). These results suggest that p72 might interact with receptors on cells and enter cells. This work still needs to be further proven. In this study, p72 affected F10 activity in vitro.

To test the effects of ASFV-encoded proteins on F10 expression in Huh7 cells, Huh7 cells were transfected with plasmids expressing ASFV-encoded proteins, including K205R, EP153R, E199L, p30, p54, and p72 (Figure 3B). Huh7 cells were collected at 24 h post-transfection for western blot analysis, and the supernatants were collected for ELISA detection of secreted F10. Factor X expression was detected to reveal the functional proteins that regulate the coagulation process. As a result, only one encoded protein, K205R, might upregulate F10 expression, whereas some proteins, such as EP153R, E199L, p30, p72, and p54, might downregulate F10 expression in Huh7 cells (Figure 3B). Consistent with F10 expression in cells, secretory F10 was also upregulated by K205R and downregulated by EP153R, E199L, p30, p72, and p54 (Figure 3C). Overall, several ASFV-encoded proteins can regulate the coagulation process by upregulating or downregulating the expression of F10, and different encoded proteins might have multiple functions at the F10 level.

### Proteomic analysis reveals that p72 is related to the coagulation cascade

**Figure 4:**
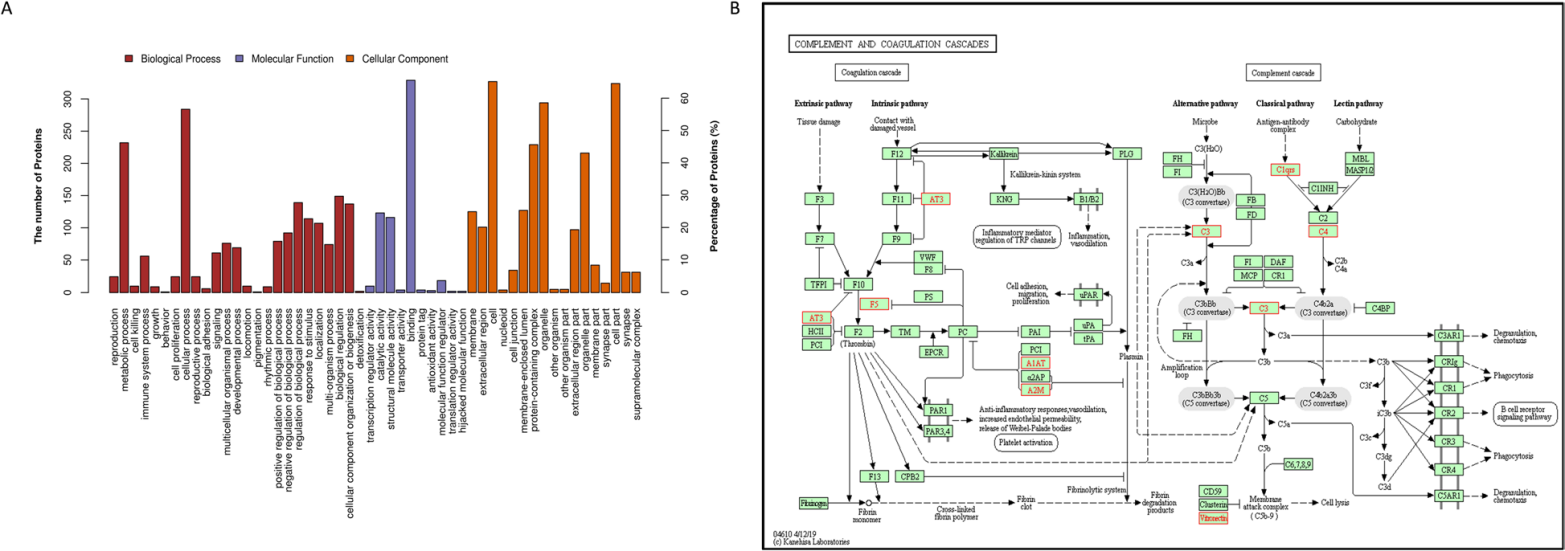
**Proteomic analysis of p72-interacting proteins**. **A** GO term analysis. Distribution of GO terms into three groups: biological process, molecular function, and cellular component. The upper coordinate represents the number of proteins; the right vertical coordinate represents the percentage of proteins. **B** Distribution of the top 20 enriched KEGG pathways. KEGG pathways of the p72-related proteins involved in coagulation and the complement cascade.

The p72-expressing plasmid of ASFV and the empty vector were transfected into 293-T cells (1 μg in each well, 12 μg in total), and the samples were prepared in triplicate. The samples of p72- and empty vector-expressing cells were collected in 1 mL of cold immunoprecipitation buffer supplemented with protease inhibitor cocktail, the lysates were cleared by centrifugation, and the supernatants were immunoprecipitated with an anti-HA antibody and then precoupled with A/G Plus agarose beads. Triplicate samples from each group were subjected to LC‒MS/MS analysis. The database obtained for empty vector-interacting proteins was deleted for p72-interacting proteins to exclude possible contaminants or to specify the proteins that interact with p72. A previous experiment identified several proteins that affect the F10 expression level. Among the encoded proteins, p72 attracted our attention. To further prove that the coagulation cascade is related to p72, proteomics analysis of p72-interacting proteins was performed. From enriched GO terms and KEGG pathway analysis, we found that complement and coagulation cascades were enriched according to the GO and KEGG analyses (Additional files 3, 4, 5, Figures 4A, B), which was consistent with our clinical coagulation tests, and the enriched genes related to the coagulation process potentially regulated by p72 are shown in Table 3. Interestingly, anticoagulation factors (anti-thrombin III) and coagulation factors (collagen alpha, factor V) were both detected, implying that the coagulation system is activated and potentially regulated to maintain balance. These findings imply that dysfunction of coagulation cascades is closely related to p72 in ASFV.

**Table 3:** **Host proteins related to the coagulation process regulated by p72**

UniProt Accession No.	Description
P01008	Antithrombin-III
P01009	Alpha-1-antitrypsin
P02452	Collagen alpha-1(I) chain
P08123	Collagen alpha-2(I) chain
P12259	Coagulation factor V
A0A2S0BDD1	Antithrombin III isoform
A0A024R944	Serpin peptidase inhibitor, clade C (Antithrombin), member 1,
Q9Y490	Talin-1
Q03113	Guanine nucleotide-binding protein subunit alpha-12
P01023	Alpha-2-macroglobulin
P19105	Myosin regulatory light chain 12A
P04004	Vitronectin
P35579	Myosin-9
P63092	Guanine nucleotide-binding protein G(s) subunit alpha isoforms
A0A0K0Q2Z1	Serpin peptidase inhibitor clade C member 1
Q07021	Complement component 1 Q subcomponent-binding protein,
P32119	Peroxiredoxin-2
V9HW12	Epididymis secretory sperm binding protein
P19075	Tetraspanin-8
P04792	Heat shock protein beta-1
P63104	14–3-3 protein zeta/delta
P60709	Actin, cytoplasmic
P52907	F-actin-capping protein subunit alpha-1
P68431	Histone H3.1

### p72 interacted with coagulation factor F10

**Figure 5:**
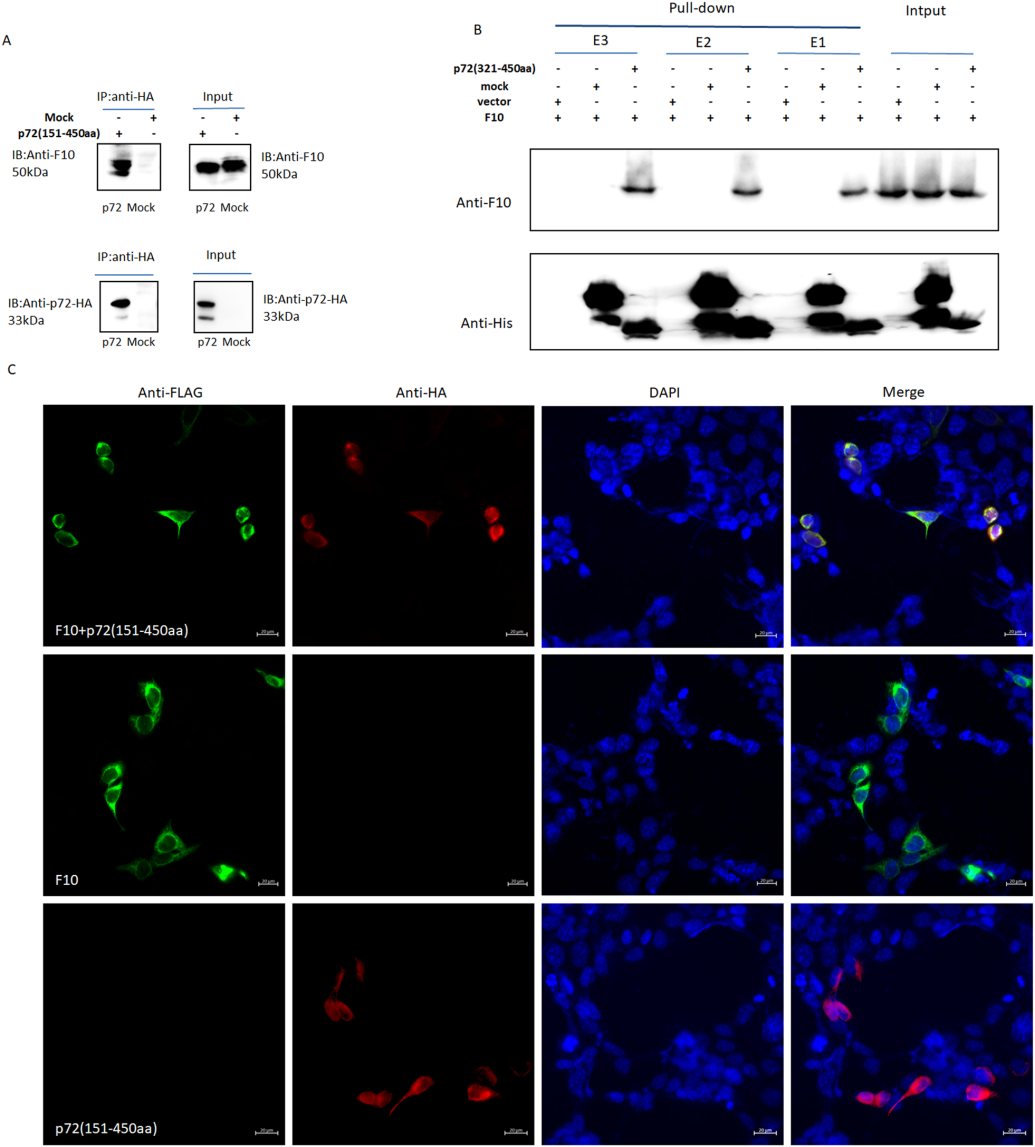
**p72 interacted with F10**. **A** 293-T cells were separately transfected with 1 μg of pcDNA3.1-p72-HA (151–450 aa) or mock plasmid for 24 h. The immune complexes were precipitated with anti-HA antibodies and subjected to western blot analysis. **B** Pull-down assay**.** p72 (321–450 aa) was secreted into *E. coli* and purified with Ni^+^ purification resin. The target and irrelevant proteins used as mock controls were mixed separately with the F10 protein. The mixtures were slowly shaken overnight at 4 °C, pulled down with Ni^+^ resin, wand anti-F10 primary antibodies were used to probe the target proteins. **C** Confocal microscopy. 293-T cells were transfected with 1 μg of pcDNA3.1-p72-HA, pcDNA3.1-F10-FLAG, or mock plasmid in 24-well culture plates for 24 h. At 24 h post-transfection, the cells were processed for confocal microscopy. The primary antibodies used were anti-HA and anti-FLAG, followed by incubation with Alexa Fluor 555-conjugated goat anti-rabbit IgG and Alexa Fluor 488-conjugated goat anti-mouse IgG (at a 1:400 dilution). The cell nuclei were stained with DAPI (blue). The co-expression of p72 (red) and F10 (green) or their independent expression levels were determined via confocal microscopy.

Previous findings indicated that the 151–450 aa fragment of p72 could inhibit F10 expression. The inhibitory effect of p72 (151–450 aa) depends on its interaction with F10. Because of their high transfection efficacy, 293-T cells were separately transfected with 1 μg of pcDNA3.1-p72-HA or mock plasmid for 24 h. The immune complexes were precipitated using anti-HA antibodies. The results of the immunoprecipitation assay indicated that p72 (151–450 aa) interacted with coagulation factor F10 (Figure 5A).

For the same reason, we prepared a p72 fragment (151–450 aa), which was not expressed in the supernatant but rather in the precipitate. However, we found that 321–450 aa were expressed in the supernatant and precipitate in the *Escherichia coli* system, which is easy to purify and potentially does not lose its biological function (Additional file 6). To test whether the shortened fragment also interacts with F10, pig F10-expressed protein, p72 protein (aa 321–aa 451) and mock (Rep protein) were prepared as previously described. The same concentrations of p72 or mock control were mixed with the F10 protein. The immune complexes were incubated overnight at 4 °C and then precoupled with 40 μL His-tag resin to immunoprecipitate the complex, and anti-F10 and anti-His antibodies were used to detect the target proteins. A pull-down assay also revealed that p72 (aa 321–450) could interact with F10 (Figure 5B) in three elutions (E1–E3), and only p72 (aa 321–450) with the F10 complex could detect both target bands. Confocal microscopy further demonstrated the colocalization of p72 and F10, which were both mainly localized in the cytoplasm and nucleus (Figure 5C). In brief, these results revealed that p72 interacted with coagulation factor F10 and that the binding domain at the N-terminus of p72 was 321–450 aa long, indicating that the binding of p72 to F10 might affect the coagulation signalling cascade.

### The N-terminal 423-450aa of p72 is the functional domain that regulates F10

**Figure 6:**
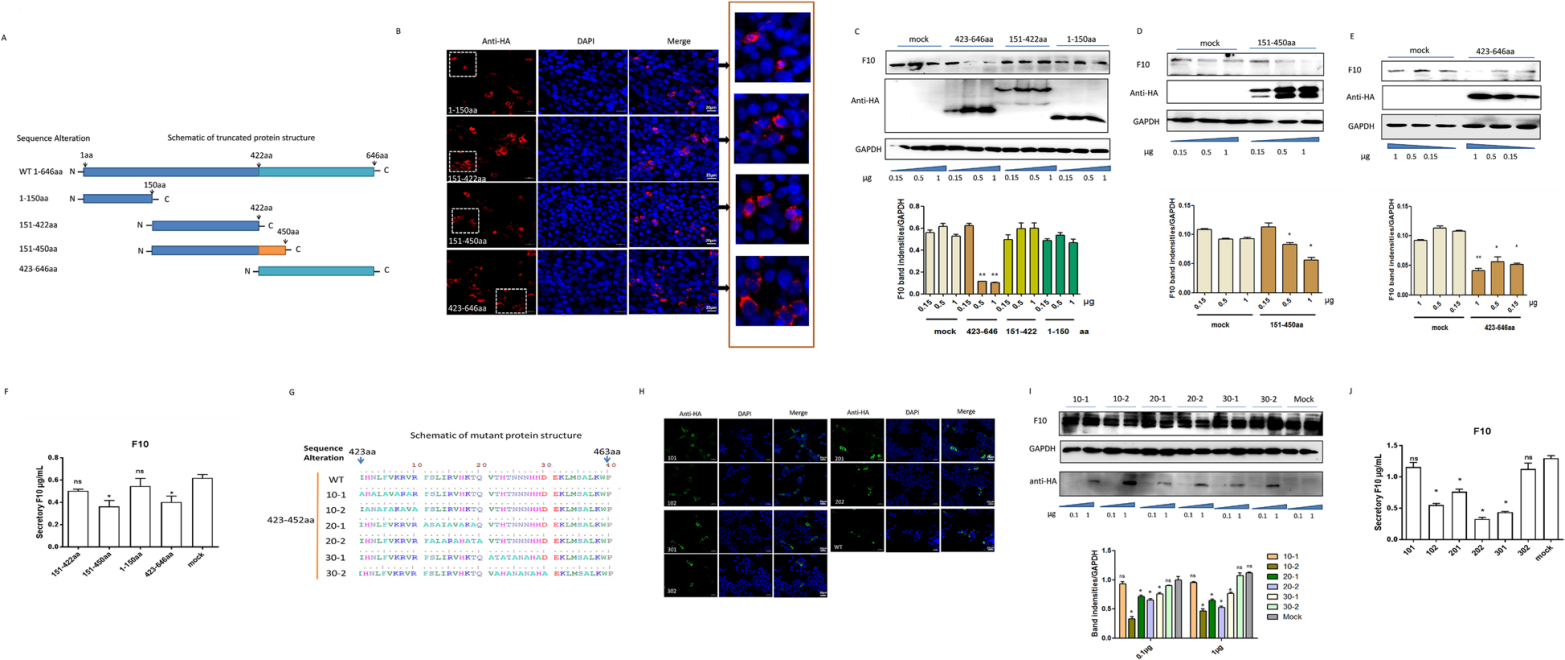
**Functional domain of p72**. **A** Schematic of truncated p72 expression. **B** Confocal microscopy of truncated proteins in Huh7 cells; the target proteins are stained red. DAPI was used to stain the sections blue. Bar, 20 μm. **C** Truncated plasmids containing amino acids 1–150, 151–422, and 423–646, **D** 151–450, **E** and 423–646 of p72 with an HA tag at the C-terminus were transfected (0.5, 1, or 2 μg) into Huh7 cells. At 24 h post-transfection, the cell lysate was collected and subjected to western blot analysis, and the **F** cell supernatant was collected for detection of secretory F10 expression via ELISA. **G** Mutant plasmid construction schematic. The sequences of the six p72 mutants ranged from 423–450 aa, and every 10 amino acids were randomly mutated to alanine, which were named 101, 102 (423–432 aa), 201, 202 (433–442 aa), 301, and 302 (443–452 aa). **H** Confocal microscopy of mutant proteins in Huh7 cells. The target proteins are stained green. DAPI was used to stain the sections blue. Bar, 20 μm. **I** The mutant plasmids were transfected into Huh7 cells at increasing doses (0.5, 1, and 2 μg)**.** At 24 h post-transfection, the cells were lysed with lysis buffer, and 15 μg of each sample was subjected to western blotting. The band intensities were normalized to those of GAPDH via ImageJ software. The data are expressed as the mean intensity ratio ± SD of F10 to GAPDH. The experiment was performed in triplicate, and images from three independent experiments were plotted. **J** The cell supernatant was also collected for detection of secretory F10 expression via ELISA. Statistical data were analysed by *t test* variance (*p < 0.05, ns represents nonsignificant *p* > 0.05).

To further confirm the functional domain of p72 with F10, we analysed the hydrophilicity and hydrophobicity of the p72 protein using the ExPASy tool (Additional file 7A). The results indicated that amino acids 1–150 at the N-terminus of p72 are potentially hydrophobic and that amino acids 151–450 have a wide range of hydrophilicities and can be highly expressed in cells. Additionally, according to the prediction of the online tool InterPro, a protein consisting of 423–646 amino acids was predicted to be a large eukaryotic DNA virus major capsid protein (Additional file 7B), also known as capsid nucleocytoplasmic large DNA virus (NCLDV), on the basis of a similar sequence. The four families of large eukaryotic DNA viruses, *Poxviridae*, *Asfarviridae*, *Iridoviridae*, and *Phycodnaviridae*, are collectively referred to as NCLDVs. Significant sequence similarity was observed between these proteins and the major capsid protein (p72) of ASFV, which also has an icosahedral capsid. Thus, four truncations were constructed and expressed, which were the 1–150, 151–422, 151–450, and 423–646 amino acid (aa) domains of p72 with an HA tag at the C-terminus. The structural schematic is shown in Figure 6A, and the truncated proteins were all expressed with subtle differences in Huh7 cells. p72 is restricted to perinuclear virus factories during infection. Without the chaperone B602L, the truncated proteins were preferentially expressed in the cytoplasm, with some scattered spots, such as those in the 1–150 aa- and 151–450 aa expressing cells (Figure 6B). Truncated p72-expressing plasmids (0.5, 1, and 2 μg) were used to overexpress p72 in Huh7 cells. The cells were lysed for western blot analysis, and the cell supernatants were collected for secretory F10 expression analysis by ELISA. As a result, 151–450 aa and 423–646 aa of p72 inhibited F10 expression, and the inhibitory effect depended on the transfection dose, while other truncated proteins failed (Figures 6C–E). The ELISA results also confirmed that the secretory F10 expression levels at 151–450 aa and 423–646 aa in p72-transfected cells were markedly lower than those in the other truncation-transfected cells (Figure 6F). Overall, the functional domain of p72 to F10 ranged from 423–450 aa.

To further refine the functional domain of p72 with F10, we constructed a p72 mutant. The mutant domains ranged from 423–450 aa in length in p72, and every 10 amino acids were randomly mutated to alanine. A schematic of the structure is shown in Figure 6G. The mutant proteins were all expressed with similar distributions in the cytoplasm of Huh7 cells (Figure 6H). The mutant plasmids were transfected into Huh7 cells at increasing doses (0.5, 1, and 2 μg), the cells were lysed for western blot analysis, and the cell supernatants were collected for secretory F10 expression detection by ELISA at 24 h post-transfection. Only the 20–1 and 30–2 mutants abolished the inhibitory effect on F10 expression according to western blot analysis (Figure 6I). The ELISA results revealed that the secretory F10 expression in the mutant p72 plasmid-transfected cells, with the exception of the 20–1- and 30–2–transfected cells, was obviously lower than that in the mock-transfected cells (Figure 6J), indicating that the key binding domains were between 423–432 aa and 443–452 aa.

### TRIM28 interacts with F10 and p72

**Figure 7:**
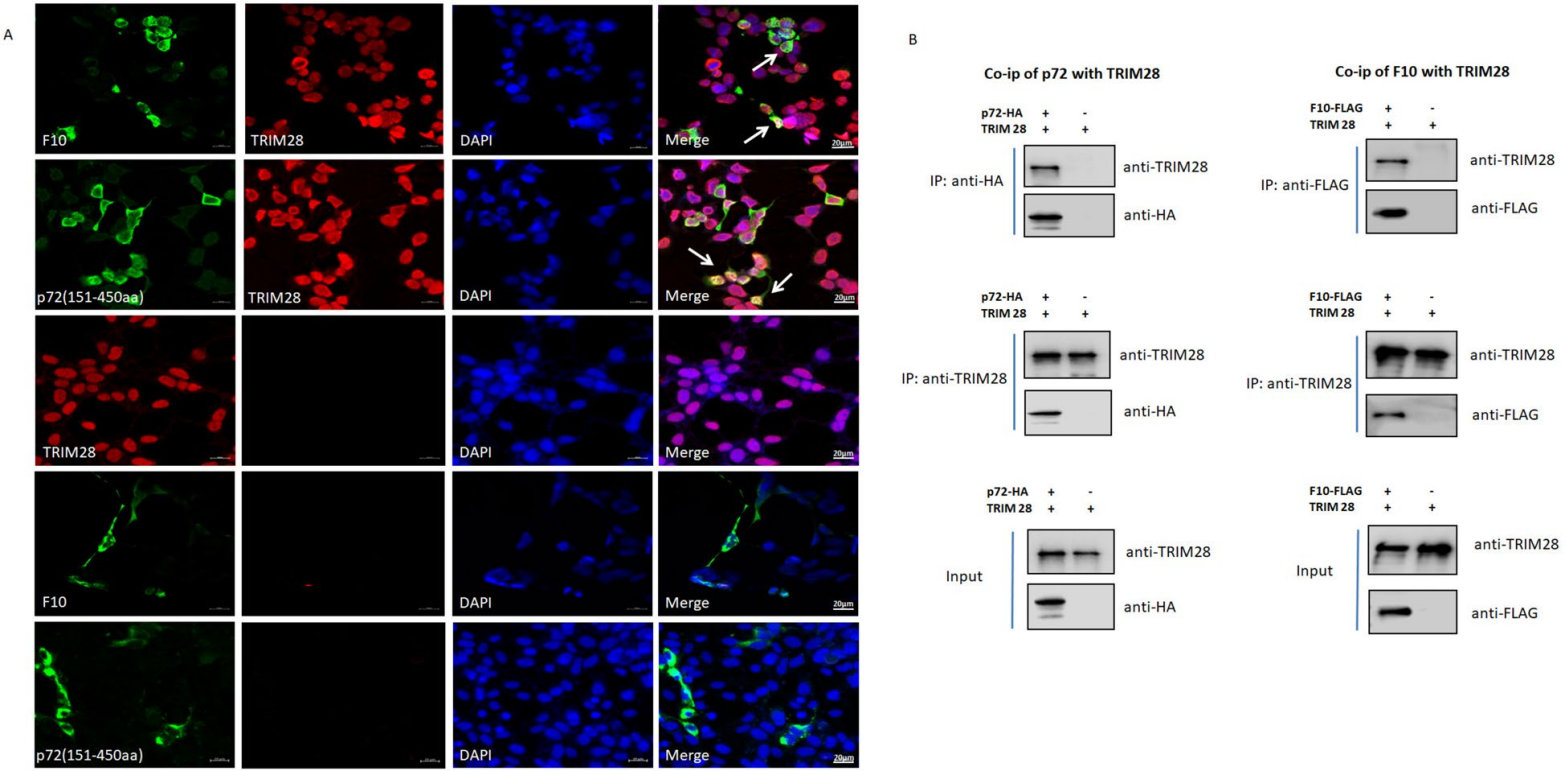
**TRIM28 interacts with p72 and F10**. **A** Co-localization of TRIM28 with p72 and F10. 293-T cells were co-transfected with p72 and F10-expressing plasmids or their respective plasmids, after which the transfected cells were immunostained. The cells were fixed and visualized by confocal microscopy. p72-HA and F10-FLAG were stained green, and TRIM28 was stained red. Scale bars, 20 μm. **B** Co-IP of TRIM28 with p72 and F10. Anti-p72-HA antibody affinity purification and anti-TRIM28 antibody affinity purification with lysates from replicon cells are indicated. The bands were quantified and normalized to the input; anti-F10-FLAG antibody affinity purification and anti-TRIM28 antibody affinity purification with lysates from the replicon cells are indicated. The bands were quantified and normalized to the input.

TRIM28 is an interacting partner of p72, as shown in Additional file 5. To determine the role of TRIM28 in the reduction in F10 by p72, confocal microscopy was performed, and the results indicated that p72 and F10 colocalized with TRIM28 in the nucleus. Moreover, TRIM28 and F10 were preferentially expressed in the cytoplasm, indicating that TRIM28 may interact with p72 and F10 in the nucleus (Figure 7A). Co-immunoprecipitation (IP) assays confirmed that TRIM28 could interact with p72 and F10 (Figure 7B). TRIM28 is expressed and functions in the nucleus, demonstrating that TIM28 might play a role in F10 degradation regulated by p72.

### TRIM28 is involved in p72-regulated F10 degradation

**Figure 8:**
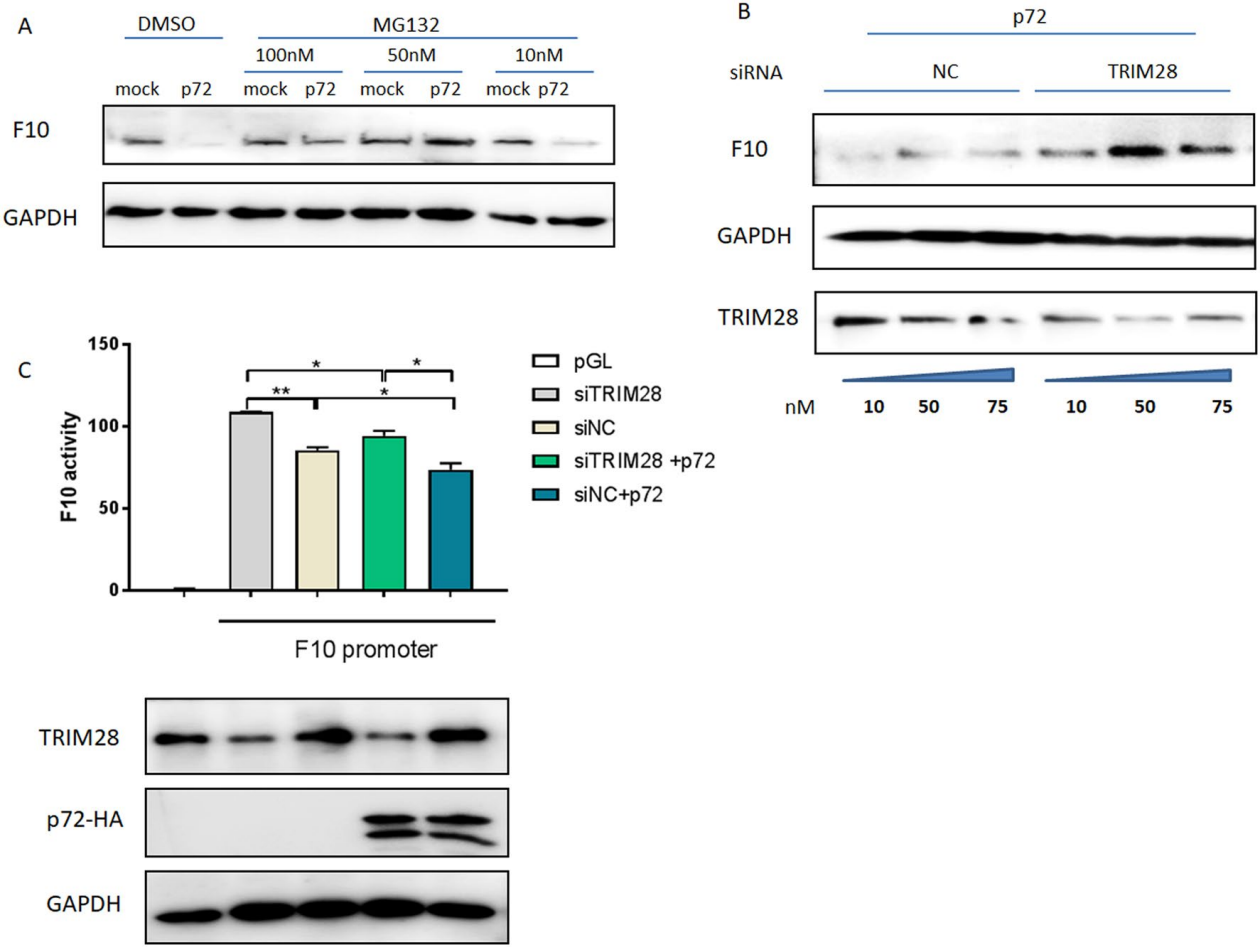
**TRIM28 is involved in F10 degradation regulated by p72**. **A** MG132 is an inhibitor of the ubiquitin‒proteasome system and was pretreated with MG132 at concentrations of 10, 50, and 100 nM for 4 h prior to p72 truncation (151–450 aa) transfection. At 24 h post-transfection, the cells were collected for western blot analysis for F10 expression. **B** siRNAs targeting TRIM28 and the NC were transfected into Huh7 cells at different concentrations (10, 50, and 75 nM). At 12 h post-transfection, the supernatant was removed, the medium was replaced with fresh medium, and the cells were then transfected with p72 truncations or mock controls (1 μg). At 24 h post-transfection with p72 truncations, the cell lysates were collected and subjected to western blot analysis. **C** siRNAs targeting TRIM28 and the NC were transfected into Huh7 cells (50 nM). At 12 h post-transfection, the supernatant was removed, replaced with fresh media, and then co-transfected with the pGL-F10 promoter plasmid (1 μg) or pRL-TK (0.1 μg) with or without p72 (151–450 aa) (1 μg). At 24 h post-transfection with the promoters, the cell lysates were collected and subjected to western blot and luciferase activity analyses. For all figures, the experiments were repeated at least three times with similar results. The data are presented as the mean ± SD from one single experiment. Statistical significance was determined by Student’s *t* test (*p < 0.05; **p < 0.01).

MG132 is an inhibitor of the ubiquitin‒proteasome system. After pretreatment with MG132 at 10, 50, or 100 nM prior to p72 truncation, the inhibitory effect of p72 on F10 expression was weakened, indicating that p72 truncation inhibited F10 via the ubiquitin‒proteasome pathway (Figure 8A). To determine the role of TRIM28 in p72-mediated regulation of F10 protein expression, we knocked down TRIM28 following p72 truncation in Huh7 cells and found that TRIM28 had no inhibitory effect on the F10 protein level, indicating that TRIM28 mediates the process by which p72 inhibits F10 expression (Figure 8B). Factor X activity was also determined after RNA interference of TRIM28 and overexpression of p72 in Huh7 cells. Compared with siNC, siTRIM28 increased F10 promoter activity, whereas p72 overexpression downregulated F10 activity in siTRIM28-treated cells, suggesting that TRIM28 and p72 are involved in inhibiting F10 activity. Surprisingly, p72 also reduced activity in TRIM28-knockdown cells, suggesting that other procoagulants and anticoagulants may also participate in regulating the activity of F10. TRIM28 can assist p72 in downregulating F10 expression but is not essential for p72-mediated decreases in F10 activity. Furthermore, the tendencies of F10 normal expression and its active form are not the same in clinical trials (Figure 1) or clinical samples (data not shown), indicating that there might be different mechanisms involved in regulating the activity or expression of F10 (Figure 8C). Collectively, these results suggest that TRIM28 may be an intermediate protein that functions as a ubiquitin E3 ligase and is associated with a decrease in F10 expression regulated by p72.

## Discussion

The ASFV pandemic has become a public crisis for the pig industry. To date, no effective vaccine against ASFV has been developed because of the complexity of the virus and the limited understanding of ASFV virulence genes. Previous studies have shown that ASFV infection can decrease thrombin and platelet counts and, ultimately, DIC in the clinic [38]. The primary symptom of ASFV infection is multiple-organ bleeding, which is the major cause of high mortality in pigs [61], but the mechanism of ASFV infection-induced coagulation is not understood. Typically, coagulation involves a balance between procoagulant and anticoagulant mechanisms. Tissue factor is a major activator of coagulation [62]. Direct or indirect activation of the endothelium by viruses or other pathogens may lead to alterations in coagulation and fibrinolytic systems [63]. The regulated activation of coagulation is part of the host’s defence against infectious agents.

D-dimer is a soluble fibrin degradation product of thrombi generated by the fibrinolytic system and serves as a valuable marker of coagulation and fibrinolysis activation for diagnosing and monitoring DIC [64, 65]. Factor II is also known as prothrombin, the precursor state of thrombin. Factor X is also a crucial coagulation factor at which extrinsic and intrinsic coagulation factors converge [21]. Elevated levels of Factor XIa-C1-inhibitor complexes were found in patients with dengue haemorrhagic fever, indicating the activation of coagulation [36]. In addition, decreasing levels of Factor VIIa were detected in macaques infected with Ebola virus [66]. Thus, the coagulation related factors were the markers of the state of coagulation and fibrinolysis system. In our study, it was unexpected that F10 activity and F2 expression did not significantly change in ASFV-infected serum. Nevertheless, D2D and F10 expression in ASFV-infected serum was significantly lower than that in noninfected serum at 0 dpi, implying that ASFV could regulate the coagulation signalling cascade by altering the expression of key factors and might cause coagulation disorders, which is consistent with clinical symptoms. Infection with ASFV increased the expression of all coagulation factors or their related factors at the initial infection. After a short period of time, the expression of these factors soon gradually decreases with increasing severity and worsening of the disease. Among these detected factors, F10 and D2D expression alterations were significant. These findings suggest that the coagulation pathway is similar to the interferon signalling pathway. The virus builds its infection upon the host and elevates multiple interferons at the beginning of the infection, while the virus develops several mechanisms to evade interferon signalling [67, 68]. The African swine fever virus might invent a similar mechanism for the coagulation process, or the deterioration of disease or tremendous depletion of coagulation factors can cause the body to become dysfunctional; thus, the host cannot produce enough coagulation factors to initiate the coagulation process.

Next, we determined that ASFV infection affects both extrinsic and intrinsic coagulation pathways by detecting F10 after RNA interference of each coagulation factor in these two pathways following ASFV infection. Nevertheless, interestingly, the expression of the intrinsic coagulation proteins F9 and F12 significantly increased at 24 and 36 hpi, whereas the expression of the extrinsic coagulation proteins F3 (TF) and F7 was not obviously different between infected and noninfected cells. Additionally, infection with different ASFV strains resulted in different results: recombinant ASFV (with the MGF360–9 L deletion gene) upregulated F3 (TF) and F7 expression but not F9 or F12 expression, which suggests that there is a complementary mechanism for the activation of coagulation cascades (Additional file 8). Similar to F9 and F12, F10 expression did not increase. In contrast, the expression of this protein decreased later after infection with a wild-type strain or recombinant virus, suggesting that the coagulation process requires considerable depletion of F10 or that ASFV infection inhibits F10 expression to inhibit the coagulation process. Our study revealed that ASFV infection could significantly reduce F10 expression in the serum of infected pigs. Consistent with the findings of the in vivo study, F10 was downregulated by ASFV infection at 36 hpi in vitro.

Surprisingly, we identified several viral proteins that affect F10 expression. The structural proteins p30, p54, and p72 are important components of viral particles that play roles in attachment, entrance, and reproduction [69]. E199L is involved in viral entry [56], and p153R is a membrane protein with a C-type animal lectin-like domain and a cell attachment sequence that participates in viral hemadsorption [60]. This finding potentially suggests that the viral proteins associated with viral attachment or invasion might decrease the expression of coagulation crucial factor 10; however, the mechanism is unknown and needs to be further studied. Proteomic analysis of p72-precipitated proteins further confirmed that p72 is involved in the coagulation cascade. Additionally, the baculovirus system was used to co-express p72 and pB602L because the p72 protein in the correct folding conformation needed the help of B602L. The co-expressed proteins p72 and pB602L inhibited F10 activity in vitro because of their ability to enter the cells [70]. The correct conformation or entry of p72 into cells might be necessary for p72 to regulate the coagulation cascade, but the precise mechanism is not understood. p72 affects the coagulation process by altering the expression and activity of F10. We also identified several proteins that did not influence F10 expression, such as p22 and pE248R (data not shown). Given that several target proteins have been found to affect F10 expression, these findings might shed light on the pathogenesis mechanism of ASFV.

In addition, we investigated the relationship between F10 and p72. In some studies, F10 could bind the structural proteins of the virus to facilitate attachment and entry into cells. For example, F10 has been proposed to act as a bridge between host heparan sulphate proteoglycans and human adenovirus major capsid protein hexon trimers by binding to hypervariable regions of hexon [71, 72]. Glycoprotein C of the herpes simplex virus binds and promotes the activation of F10 on infected endothelial cells, thereby contributing to thrombin generation [73]. Thus, we tested whether F10 could regulate ASFV replication. However, F10 activity was not involved in ASFV replication when an F10-specific inhibitor was used (Additional file 9). The precise relationship between F10 and ASFV needs to be further studied.

Mechanistically, in an attempt to confirm the binding domain of p72 with F10, we defined 423–450 aa as the most important functional domain by constructing and expressing truncated and mutant p72 expression plasmids. Given that p72 can downregulate F10 protein expression, we investigated whether this inhibitory effect depends on the ubiquitin‒proteasome pathway. When cells were treated with MG132, a small molecule inhibitor of the ubiquitin‒proteasome pathway, the degradation effect on F10 expression induced by p72 was alleviated, suggesting that p72 modulates F10 via the proteasomal degradation pathway. Next, p72-interacting partners involved in proteasome pathways were identified, and TRIM28 attracted our attention; the protein functions as an E3 ubiquitin ligase via its RING domain. To the best of our knowledge, F10 and p72 colocalize with TRIM28 in the nucleus, where TRIM28 is expressed and functional. These co-IP results are consistent with the confocal microscopy results showing that F10 and p72 interact with TRIM28, suggesting that TRIM28 might play a role in the p72-mediated reduction in F10 protein expression. The possible reason is that p72 recruits TRIM28 to degrade F10, and knocking down TRIM28 counteracts the reduction in F10 expression. In addition, F10 activity was upregulated at the protein level by RNA interference of TRIM28 and downregulated by p72 overexpression. However, whether the activity of F10 is associated with its expression level has not been tested; it is known that p72 cooperates with TRIM28 to affect F10 expression and activity. TRIM28, a p72-binding protein, contributes to p72 functional regulation, thereby leading to subsequent F10 proteasomal degradation.

Overall, for the first time, it was reported that infection with ASFV and its encoded proteins can regulate the coagulation process by reducing F10 expression both in vitro and in vivo. In addition, the major capsid protein p72 can interact with F10 and inhibit the coagulation cascade induced by ASFV infection. Notably, TRIM28 plays an important role in mediating the degradation of F10 regulated by p72. Our findings highlight the multifaceted complexity of virus‒host protein interactions and reveal a novel mechanism by which ASFV regulates the coagulation process.

## Supplementary Information

**Additional file 1**. **siRNA fragment selection**. Three siRNA fragments of F1-F12 and control siRNA were transfected into 3D4 and PIEC at 1 μg. At 24 h post-transfection, the cell lysates were collected for western blot analysis and probed with the corresponding primary antibodies.

**Additional file 2**. **Entering into cells of Baculovirus co-expressed p72 and B602L**. Baculovirus coexpressing p72 and B602Lwas added to the PK-cell culture for 0 min, 15 min, 30 min, 45 min and 1 h. Then, the cells at the indicated time points were fixed and probed with an anti-p72 antibody, and the results are shown with green fluorescence. The cell borders were stained red with phalloidin.

**Additional file 3**. **GO terms and corresponding proteins of p72-interacting partners**.

**Additional file 4**. **KEGG pathways and corresponding proteins associated with p72-interacting partners**.

**Additional file 5**. **Protein accession numbers and descriptions of p72-interacting partners**.

**Additional file 6**. **Prokaryotic expression of p72**. **A** Truncated p72 and the vector were expressed in the prokaryotic expression system, and the supernatant and precipitates were collected for SDS‒PAGE analysis. The black arrows indicate the target bands. **B** Supernatants and p72 and vector precipitates were also analysed by western blotting. A primary antibody against His was used to probe the target bands.

**Additional file 7**. **Hydrophilicity, hydrophobicity and functional domain analysis of p72**. **A** The hydrophilicity and hydrophobicity of p72 were analysed with the ExPASy online tool to determine the potential highly expressed domain. **B** The functional domain of p72 was predicted by the InterPro online tool, and functional domain prediction was based on its sequence similarity with other NCLDV family members. The boundaries of the truncations are indicated by the arrows.

**Additional file 8**. **ASFV recombinant strain infection regulated coagulation factor expression**. PAM cells were infected with 0.1 MOI ASFV for 6, 12, 24 or 36 h. Intrinsic coagulation factors, crucial coagulation factor F10 and extrinsic coagulation factors were detected by western blotting at the indicated time points.

**Additional file 9**. **F10 activity was not involved in the replication of ASFV**. F10-specific inhibitors at increasing doses were used to test the role of F10 in ASFV replication; PAMs were treated with F10 inhibitors prior to and during ASFV infection. The viral loads of the supernatants and cell lysates were detected via qPCR at 36 hpi. Statistical data were analysed by *t* test analysis of variance. All the data are expressed as the mean ± SD.

## Data Availability

The data are contained within the manuscript and supporting materials.

## Abbreviations

ASFV: African swine fever
F10: factor x
qRT-PCR: quantitative RT-PCR
PAM: porcine alveolar macrophage
D2D: D-dimer
TRIM28: tripartite motif-containing protein 28
HAD50: 50% Hemadsorption dose
GAPDH: glyceraldehyde-3-phosphate dehydrogenase
MOI: multiplicity of infection
LC‒MS/MS: liquid chromatography–mass spectrometry
ELISA: enzyme-linked immunosorbent assay

## References

[1] Dixon LK, Sun H, Roberts H (2019) African swine fever. Antiviral Res 165:34–41 30836106 10.1016/j.antiviral.2019.02.018

[2] Ge S, Li J, Fan X, Liu F, Li L, Wang Q, Ren W, Bao J, Liu C, Wang H, Liu Y, Zhang Y, Xu T, Wu X, Wang Z (2018) Molecular characterization of African swine fever virus, China, 2018. Emerg Infect Dis 24:2131–2133 30141772 10.3201/eid2411.181274 PMC6199985

[3] Zhou X, Li N, Luo Y, Liu Y, Miao F, Chen T, Zhang S, Cao P, Li X, Tian K, Qiu HJ, Hu R (2018) Emergence of African swine fever in China, 2018. Transbound Emerg Dis 65:1482–1484 30102848 10.1111/tbed.12989

[4] You SB, Liu TY, Zhang M, Zhao X, Dong YZ, Wu B, Wang YZ, Li J, Wei XJ, Shi BF (2021) African swine fever outbreaks in China led to gross domestic product and economic losses. Nat Food 2:802–808 37117973 10.1038/s43016-021-00362-1

[5] Salas ML, Andres G (2013) African swine fever virus morphogenesis. Virus Res 173:29–41 23059353 10.1016/j.virusres.2012.09.016

[6] Dixon LK, Chapman DA, Netherton CL, Upton C (2013) African swine fever virus replication and genomics. Virus Res 173:3–14 23142553 10.1016/j.virusres.2012.10.020

[7] Revilla Y, Perez-Nunez D, Richt JA (2018) African swine fever virus biology and vaccine approaches. Adv Virus Res 100:41–74 29551143 10.1016/bs.aivir.2017.10.002

[8] Gomez-Villamandos JC, Bautista MJ, Sanchez-Cordon PJ, Carrasco L (2013) Pathology of African swine fever: the role of monocyte-macrophage. Virus Res 173:140–149 23376310 10.1016/j.virusres.2013.01.017

[9] Galindo I, Alonso C (2017) African swine fever virus: a review. Viruses 9:103 28489063 10.3390/v9050103 PMC5454416

[10] Urbano AC, Ferreira F (2022) African swine fever control and prevention: an update on vaccine development. Emerg Microbes Infect 11:2021–2033 35912875 10.1080/22221751.2022.2108342 PMC9423837

[11] Chen WY, Zhao DM, He XJ, Liu RQ, Wang ZL, Zhang XF, Li F, Shan D, Chen HF, Zhang JW, Wang LL, Wen ZY, Wang XJ, Guan YT, Liu JX, Bu ZG (2020) A seven-gene-deleted African swine fever virus is safe and effective as a live attenuated vaccine in pigs. Sci China Life Sci 63:623–634 32124180 10.1007/s11427-020-1657-9 PMC7223596

[12] Tran XH, Le TTP, Nguyen QH, Do TT, Nguyen VD, Gay CG, Borca MV, Gladue DP (2022) African swine fever virus vaccine candidate ASFV-G-DeltaI177L efficiently protects European and native pig breeds against circulating Vietnamese field strain. Transbound Emerg Dis 69:e497–e504 34582622 10.1111/tbed.14329

[13] Tran XH, Phuong LTT, Huy NQ, Thuy DT, Nguyen VD, Quang PH, Ngon QV, Rai A, Gay CG, Gladue DP, Borca MV (2022) Evaluation of the safety profile of the ASFV vaccine candidate ASFV-G-DeltaI177L. Viruses 14:896 35632638 10.3390/v14050896 PMC9147362

[14] Neser JA, Kotze C (1987) African swine fever. II. Functional disturbances of thrombocytes in pigs infected with virulent haemadsorbing and non-haemadsorbing virus isolates. Onderstepoort J Vet Res 54:147–155 3627730

[15] Rodriguez F, Fernandez A, Martin de las Mulas JP, Sierra MA, Jover A (1996) African swine fever: morphopathology of a viral haemorrhagic disease. Vet Rec 139:249–254 8888559 10.1136/vr.139.11.249

[16] Zhao DM, Sun EC, Huang LY, Ding LL, Zhu YM, Zhang JW, Shen DD, Zhang XF, Zhang ZJ, Ren T, Wang W, Li F, He XJ, Bu ZG (2023) Highly lethal genotype I and II recombinant African swine fever viruses detected in pigs. Nat Commun 14:3096 37248233 10.1038/s41467-023-38868-w PMC10226439

[17] Villeda CJ, Williams SM, Wilkinson PJ, Vinuela E (1993) Consumption coagulopathy associated with shock in acute African swine fever. Arch Virol 133:467–475 8257301 10.1007/BF01313784

[18] Zhao D, Liu R, Zhang X, Li F, Wang J, Zhang J, Liu X, Wang L, Zhang J, Wu X, Guan Y, Chen W, Wang X, He X, Bu Z (2019) Replication and virulence in pigs of the first African swine fever virus isolated in China. Emerg Microbes Infect 8:438–447 30898043 10.1080/22221751.2019.1590128 PMC6455124

[19] Opal SM, Esmon CT (2003) Bench-to-bedside review: functional relationships between coagulation and the innate immune response and their respective roles in the pathogenesis of sepsis. Crit Care 7:23–38 12617738 10.1186/cc1854 PMC154114

[20] Esmon CT (2004) Interactions between the innate immune and blood coagulation systems. Trends Immunol 25:536–542 15364056 10.1016/j.it.2004.08.003 PMC7185622

[21] Levi M, Keller TT, van Gorp E, ten Cate H (2003) Infection and inflammation and the coagulation system. Cardiovasc Res 60:26–39 14522404 10.1016/s0008-6363(02)00857-x

[22] Levi M, van der Poll T (2010) Inflammation and coagulation. Crit Care Med 38:S26-34 20083910 10.1097/CCM.0b013e3181c98d21

[23] Dahlback B (2005) Blood coagulation and its regulation by anticoagulant pathways: genetic pathogenesis of bleeding and thrombotic diseases. J Intern Med 257:209–223 15715678 10.1111/j.1365-2796.2004.01444.x

[24] Kenne E, Nickel KF, Long AT, Fuchs TA, Stavrou EX, Stahl FR, Renne T (2015) Factor XII: a novel target for safe prevention of thrombosis and inflammation. J Intern Med 278:571–585 26373901 10.1111/joim.12430

[25] Nemerson Y, Repke D (1985) Tissue factor accelerates the activation of coagulation factor VII: the role of a bifunctional coagulation cofactor. Thromb Res 40:351–358 4082113 10.1016/0049-3848(85)90270-1

[26] Amara U, Rittirsch D, Flierl M, Bruckner U, Klos A, Gebhard F, Lambris JD, Huber-Lang M (2008) Interaction between the coagulation and complement system. Adv Exp Med Biol 632:71–79 19025115 10.1007/978-0-387-78952-1_6 PMC2713875

[27] Palta S, Saroa R, Palta A (2014) Overview of the coagulation system. Indian J Anaesth 58:515–523 25535411 10.4103/0019-5049.144643 PMC4260295

[28] Weisel JW (2005) Fibrinogen and fibrin. Adv Protein Chem 70:247–299 15837518 10.1016/S0065-3233(05)70008-5

[29] Boral BM, Williams DJ, Boral LI (2016) Disseminated intravascular coagulation. Am J Clin Pathol 146:670–680 28013226 10.1093/ajcp/aqw195

[30] Crum-Cianflone NF, Weekes J, Bavaro M (2008) Review: thromboses among HIV-infected patients during the highly active antiretroviral therapy era. AIDS Patient Care STDS 22:771–778 18783326 10.1089/apc.2008.0010 PMC2753452

[31] Keller TT, van der Sluijs KF, de Kruif MD, Gerdes VE, Meijers JC, Florquin S, van der Poll T, van Gorp EC, Brandjes DP, Buller HR, Levi M (2006) Effects on coagulation and fibrinolysis induced by influenza in mice with a reduced capacity to generate activated protein C and a deficiency in plasminogen activator inhibitor type 1. Circ Res 99:1261–1269 17068293 10.1161/01.RES.0000250834.29108.1a

[32] Violi F, Ferro D, Basili S, Artini M, Valesini G, Levrero M, Cordova C (1995) Increased rate of thrombin generation in hepatitis C virus cirrhotic patients. Relationship to venous thrombosis. J Investig Med 43:550–554 8605614

[33] Bray M, Hatfill S, Hensley L, Huggins JW (2001) Haematological, biochemical and coagulation changes in mice, guinea-pigs and monkeys infected with a mouse-adapted variant of Ebola Zaire virus. J Comp Pathol 125:243–253 11798241 10.1053/jcpa.2001.0503

[34] Feffer SE, Fox RL, Orsen MM, Harjai KJ, Glatt AE (1995) Thrombotic tendencies and correlation with clinical status in patients infected with HIV. South Med J 88:1126–1130 7481983 10.1097/00007611-199511000-00008

[35] Mairuhu AT, Mac Gillavry MR, Setiati TE, Soemantri A, ten Cate H, Brandjes DP, van Gorp EC (2003) Is clinical outcome of dengue-virus infections influenced by coagulation and fibrinolysis? A critical review of the evidence. Lancet Infect Dis 3:33–41 12505032 10.1016/s1473-3099(03)00487-0

[36] van Gorp EC, Minnema MC, Suharti C, Mairuhu AT, Brandjes DP, ten Cate H, Hack CE, Meijers JC (2001) Activation of coagulation factor XI, without detectable contact activation in dengue haemorrhagic fever. Br J Haematol 113:94–99 11328287 10.1046/j.1365-2141.2001.02710.x

[37] Edwards JF, Dodds WJ, Slauson DO (1985) Mechanism of thrombocytopenia in African swine fever. Am J Vet Res 46:2058–2063 4062007

[38] Zakaryan H, Karalova E, Voskanyan H, Ter-Pogossyan Z, Nersisyan N, Hakobyan A, Saroyan D, Karalyan Z (2014) Evaluation of hemostaseological status of pigs experimentally infected with African swine fever virus. Vet Microbiol 174:223–228 25239678 10.1016/j.vetmic.2014.08.029

[39] Edwards JF, Dodds WJ, Slauson DO (1984) Coagulation changes in African swine fever virus infection. Am J Vet Res 45:2414–2420 6441489

[40] Montaner-Tarbes S, Pujol M, Jabbar T, Hawes P, Chapman D, Portillo HD, Fraile L, Sanchez-Cordon PJ, Dixon L, Montoya M (2019) Serum-derived extracellular vesicles from African swine fever virus-infected pigs selectively recruit viral and porcine proteins. Viruses 11:882 31547130 10.3390/v11100882 PMC6832119

[41] Cavaillon JM, Adib-Conquy M (2005) Monocytes/macrophages and sepsis. Crit Care Med 33:S506-509 16340435 10.1097/01.ccm.0000185502.21012.37

[42] Wu C, Lu W, Zhang Y, Zhang G, Shi X, Hisada Y, Grover SP, Zhang X, Li L, Xiang B, Shi J, Li XA, Daugherty A, Smyth SS, Kirchhofer D, Shiroishi T, Shao F, Mackman N, Wei Y, Li Z (2019) Inflammasome activation triggers blood clotting and host death through pyroptosis. Immunity 50:1401-1411.e4 31076358 10.1016/j.immuni.2019.04.003 PMC6791531

[43] McGee MP, Wallin R, Wheeler FB, Rothberger H (1989) Initiation of the extrinsic pathway of coagulation by human and rabbit alveolar macrophages: a kinetic study. Blood 74:1583–1590 2790188

[44] Jiang P, Xue D, Zhang Y, Ye L, Liu Y, Makale M, Kesari S, Edgington TS, Liu C (2014) The extrinsic coagulation cascade and tissue factor pathway inhibitor in macrophages: a potential therapeutic opportunity for atherosclerotic thrombosis. Thromb Res 133:657–666 24485401 10.1016/j.thromres.2014.01.012

[45] Gando S, Levi M, Toh CH (2016) Disseminated intravascular coagulation. Nat Rev Dis Primers 2:16037 27250996 10.1038/nrdp.2016.37

[46] Hotchkiss RS, Moldawer LL, Opal SM, Reinhart K, Turnbull IR, Vincent JL (2016) Sepsis and septic shock. Nat Rev Dis Primers 2:16045 28117397 10.1038/nrdp.2016.45 PMC5538252

[47] Venturini L, You J, Stadler M, Galien R, Lallemand V, Koken MH, Mattei MG, Ganser A, Chambon P, Losson R, de Thé H (1999) TIF1gamma, a novel member of the transcriptional intermediary factor 1 family. Oncogene 18:1209–1217 10022127 10.1038/sj.onc.1202655

[48] Khetchoumian K, Teletin M, Mark M, Lerouge T, Cervino M, Oulad-Abdelghani M, Chambon P, Losson R (2004) TIF1delta, a novel HP1-interacting member of the transcriptional intermediary factor 1 (TIF1) family expressed by elongating spermatids. J Biol Chem 279:48329–48341 15322135 10.1074/jbc.M404779200

[49] Peng H, Begg GE, Schultz DC, Friedman JR, Jensen DE, Speicher DW, Rauscher FJ 3rd (2000) Reconstitution of the KRAB-KAP-1 repressor complex: a model system for defining the molecular anatomy of RING-B box-coiled-coil domain-mediated protein-protein interactions. J Mol Biol 295:1139–1162 10653693 10.1006/jmbi.1999.3402

[50] Wang C, Ivanov A, Chen L, Fredericks WJ, Seto E, Rauscher FJ 3rd, Chen J (2005) MDM2 interaction with nuclear corepressor KAP1 contributes to p53 inactivation. EMBO J 24:3279–3290 16107876 10.1038/sj.emboj.7600791 PMC1224681

[51] Czerwinska P, Mazurek S, Wiznerowicz M (2017) The complexity of TRIM28 contribution to cancer. J Biomed Sci 24:63 28851455 10.1186/s12929-017-0374-4 PMC5574234

[52] Ai Q, Lin X, Xie H, Li B, Liao M, Fan H (2021) Proteome analysis in PAM cells reveals that African swine fever virus can regulate the level of intracellular polyamines to facilitate its own replication through ARG1. Viruses 13:1236 34206713 10.3390/v13071236 PMC8310191

[53] Morton D, Bertram TA (1988) Isolation and preliminary in vitro characterization of the porcine pulmonary intravascular macrophage. J Leukoc Biol 43:403–410 3163716 10.1002/jlb.43.5.403

[54] Mirsaeidi SM, Houshmand M, Tabarsi P, Banoei MM, Zargari L, Amiri M, Mansouri SD, Sanati MH, Masjedi MR (2006) Lack of association between interferon-gamma receptor-1 polymorphism and pulmonary TB in Iranian population sample. J Infect 52:374–377 16233916 10.1016/j.jinf.2005.08.009

[55] Alejo A, Matamoros T, Guerra M, Andres G (2018) A proteomic atlas of the African swine fever virus particle. J Virol 92:e01293-18 30185597 10.1128/JVI.01293-18 PMC6232493

[56] Matamoros T, Alejo A, Rodríguez JM, Hernáez B, Guerra M, Fraile-Ramos A, Andrés G (2020) African swine fever virus protein pE199L mediates virus entry by enabling membrane fusion and core penetration. MBio 11:e00789-20 32788374 10.1128/mBio.00789-20 PMC7439464

[57] Afonso CL, Alcaraz C, Brun A, Sussman MD, Onisk DV, Escribano JM, Rock DL (1992) Characterization of P-30, a highly antigenic membrane and secreted protein of African swine fever virus. Virology 189:368–373 1604821 10.1016/0042-6822(92)90718-5

[58] Borca MV, Ramirez-Medina E, Silva E, Vuono E, Rai A, Pruitt S, Holinka LG, Velazquez-Salinas L, Zhu J, Gladue DP (2020) Development of a highly effective African swine fever virus vaccine by deletion of the I177L gene results in sterile immunity against the current epidemic Eurasia strain. J Virol 94:e2017–19 10.1128/JVI.02017-19 PMC7081903 31969432

[59] Wang Q, Zhou LY, Wang J, Su D, Li DH, Du YK, Yang GY, Zhang GP, Chu BB (2022) African swine fever virus K205R induces ER stress and consequently activates autophagy and the NF-κB signaling pathway. Viruses 14:394 35215987 10.3390/v14020394 PMC8880579

[60] Galindo I, Almazán F, Bustos MJ, Viñuela E, Carrascosa AL (2000) African swine fever virus EP153R open reading frame encodes a glycoprotein involved in the hemadsorption of infected cells. Virology 266:340–351 10639320 10.1006/viro.1999.0080

[61] Netherton CL, Connell S, Benfield CTO, Dixon LK (2019) The Genetics of life and death: virus-host interactions underpinning resistance to African swine fever, a viral hemorrhagic disease. Front Genet 10:402 31130984 10.3389/fgene.2019.00402 PMC6509158

[62] Maroney SA, Mast AE (2011) Tissue factor pathway inhibitor and bacterial infection. J Thromb Haemost 9:119–121 21210950 10.1111/j.1538-7836.2010.04111.x PMC3034136

[63] van Gorp EC, Suharti C, ten Cate H, Dolmans WM, van der Meer JW, ten Cate JW, Brandjes DP (1999) Review: infectious diseases and coagulation disorders. J Infect Dis 180:176–186 10353876 10.1086/314829

[64] Weitz JI, Fredenburgh JC, Eikelboom JW (2017) A test in context: D-Dimer. J Am Coll Cardiol 70:2411–2420 29096812 10.1016/j.jacc.2017.09.024

[65] Adam SS, Key NS, Greenberg CS (2009) D-dimer antigen: current concepts and future prospects. Blood 113:2878–2887 19008457 10.1182/blood-2008-06-165845

[66] Geisbert TW, Hensley LE, Jahrling PB, Larsen T, Geisbert JB, Paragas J, Young HA, Fredeking TM, Rote WE, Vlasuk GP (2003) Treatment of Ebola virus infection with a recombinant inhibitor of factor VIIa/tissue factor: a study in rhesus monkeys. Lancet 362:1953–1958 14683653 10.1016/S0140-6736(03)15012-X

[67] Zhu ZX, Li SS, Ma CA, Yang F, Cao WJ, Liu HA, Chen X, Feng T, Shi ZW, Tian H, Zhang KS, Chen HJ, Liu XT, Zheng HX (2023) African swine fever virus E184L protein interacts with innate immune adaptor STING to block IFN production for viral replication and pathogenesis. J Immunol 210:442–458 36602826 10.4049/jimmunol.2200357

[68] Huang Z, Cao HX, Zeng FL, Lin SZ, Chen JL, Luo Y, You JY, Kong CY, Mai ZZ, Deng J, Guo WT, Chen XN, Wang H, Zhou P, Zhang GH, Gong L (2023) African swine fever virus MGF505-7R interacts with interferon regulatory factor 9 to evade the type I interferon signaling pathway and promote viral replication. J Virol 97:e0197722 36815839 10.1128/jvi.01977-22 PMC10062159

[69] Jia N, Ou YW, Pejsak Z, Zhang YG, Zhang J (2017) Roles of African swine fever virus structural proteins in viral infection. J Vet Res 61:135–143 29978065 10.1515/jvetres-2017-0017 PMC5894393

[70] Liu Q, Ma B, Qian N, Zhang F, Tan X, Lei J, Xiang Y (2019) Structure of the African swine fever virus major capsid protein p72. Cell Res 29:953–955 31530894 10.1038/s41422-019-0232-x PMC6889146

[71] Findlay JS, Cook GP, Blair GE (2018) Blood coagulation factor X exerts differential effects on adenovirus entry into human lymphocytes. Viruses 10:20 29301346 10.3390/v10010020 PMC5795433

[72] Alba R, Bradshaw AC, Parker AL, Bhella D, Waddington SN, Nicklin SA, van Rooijen N, Custers J, Goudsmit J, Barouch DH, McVey JH, Baker AH (2009) Identification of coagulation factor (F)X binding sites on the adenovirus serotype 5 hexon: effect of mutagenesis on FX interactions and gene transfer. Blood 114:965–971 19429866 10.1182/blood-2009-03-208835 PMC2721791

[73] Etingin OR, Silverstein RL, Friedman HM, Hajjar DP (1990) Viral activation of the coagulation cascade: molecular interactions at the surface of infected endothelial cells. Cell 61:657–662 2160855 10.1016/0092-8674(90)90477-v

