# Supplementary figures and images for: TRIM28 regulates the coagulation cascade inhibited by p72 of African swine fever virus
Source: Vet Res. 2024 Nov 12;55:149. doi: 10.1186/s13567-024-01407-6 (PMC11559047; doi:10.1186/s13567-024-01407-6)

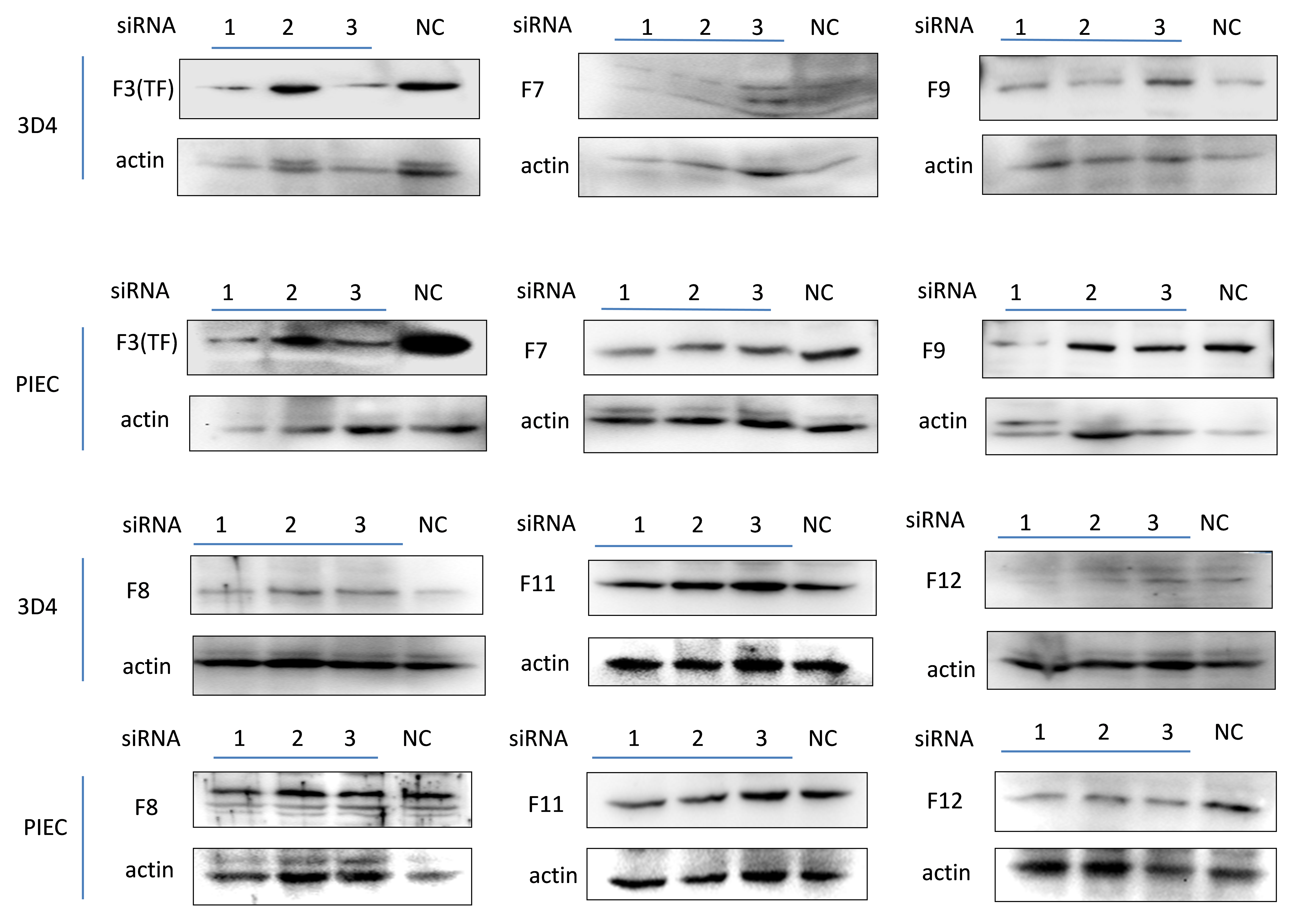

Supplement: Supplementary file 1 — Additional file 1. siRNA fragment selection. Three siRNA fragments of F1-F12 and control siRNA were transfected into 3D4 and PIEC at 1 μg. At 24 h post-transfection, the cell lysates were collected for western blot analysis and probed with the corresponding primary antibodies. [file 13567_2024_1407_MOESM1_ESM.tif]

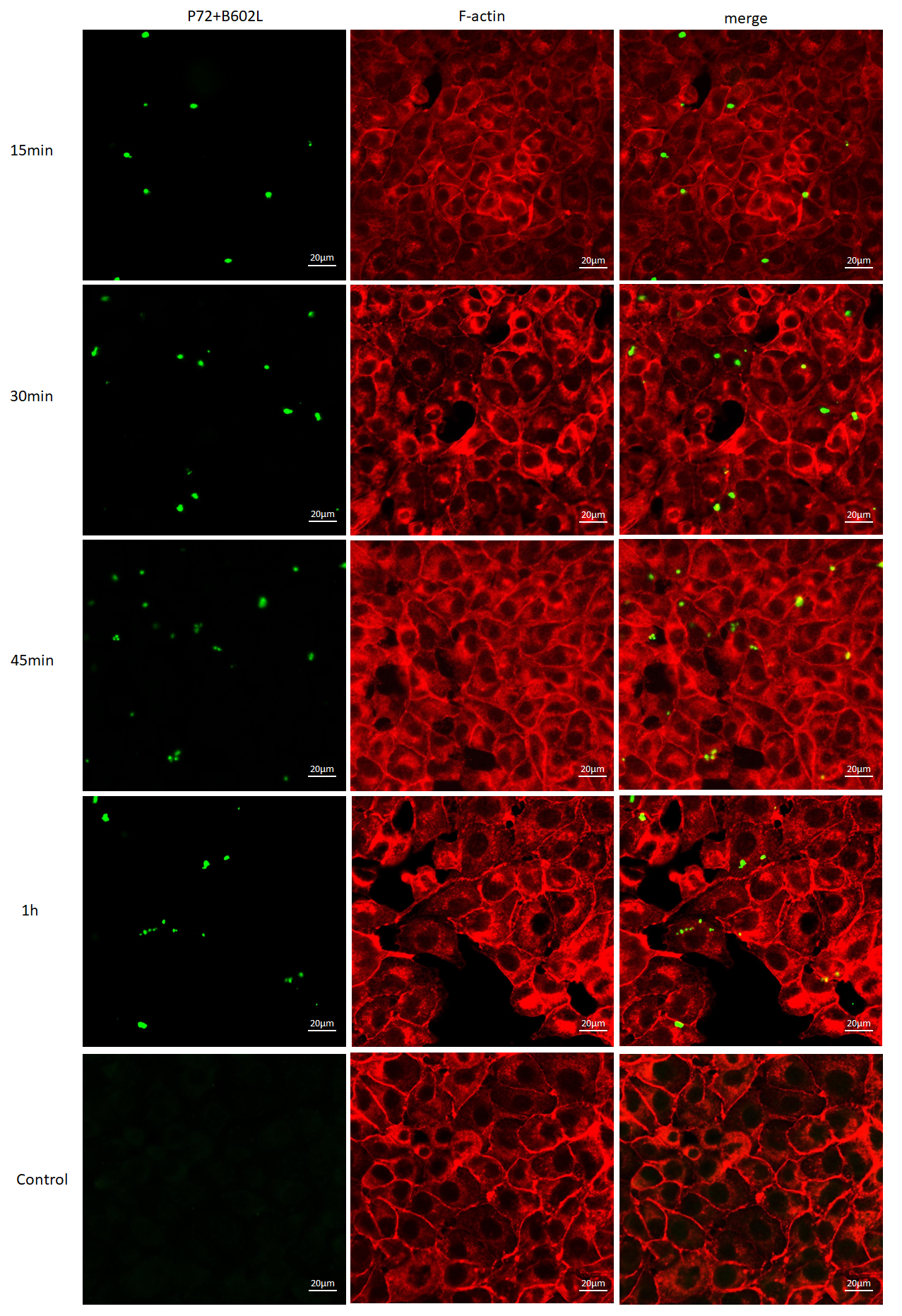

Supplement: Supplementary file 2 — Additional file 2. Entering into cells of Baculovirus co-expressed p72 and B602L. Baculovirus coexpressing p72 and B602Lwas added to the PK-cell culture for 0 min, 15 min, 30 min, 45 min and 1 h. Then, the cells at the indicated time points were fixed and probed with an anti-p72 antibody, and the results are shown with green fluorescence. The cell borders were stained red with phalloidin. [file 13567_2024_1407_MOESM2_ESM.tif]

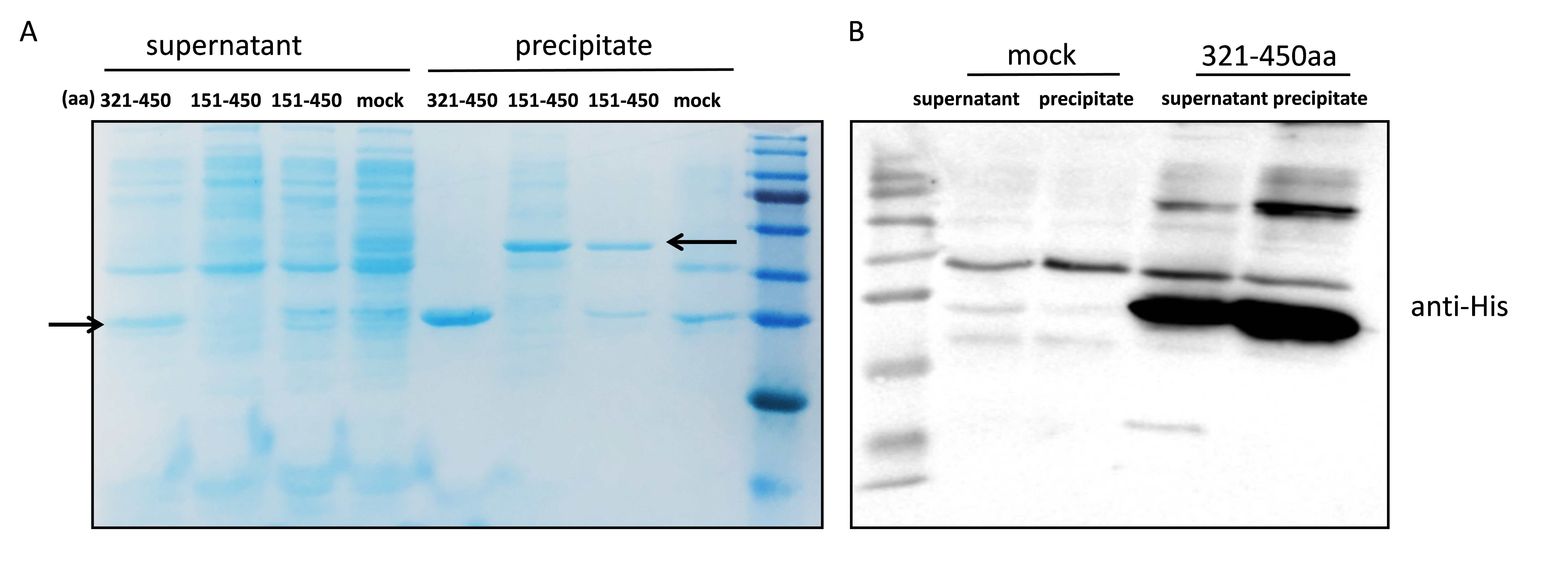

Supplement: Supplementary file 6 — Additional file 6. Prokaryotic expression of p72. A Truncated p72 and the vector were expressed in the prokaryotic expression system, and the supernatant and precipitates were collected for SDS‒PAGE analysis. The black arrows indicate the target bands. B Supernatants and p72 and vector precipitates were also analysed by western blotting. A primary antibody against His was used to probe the target bands. [file 13567_2024_1407_MOESM6_ESM.tif]

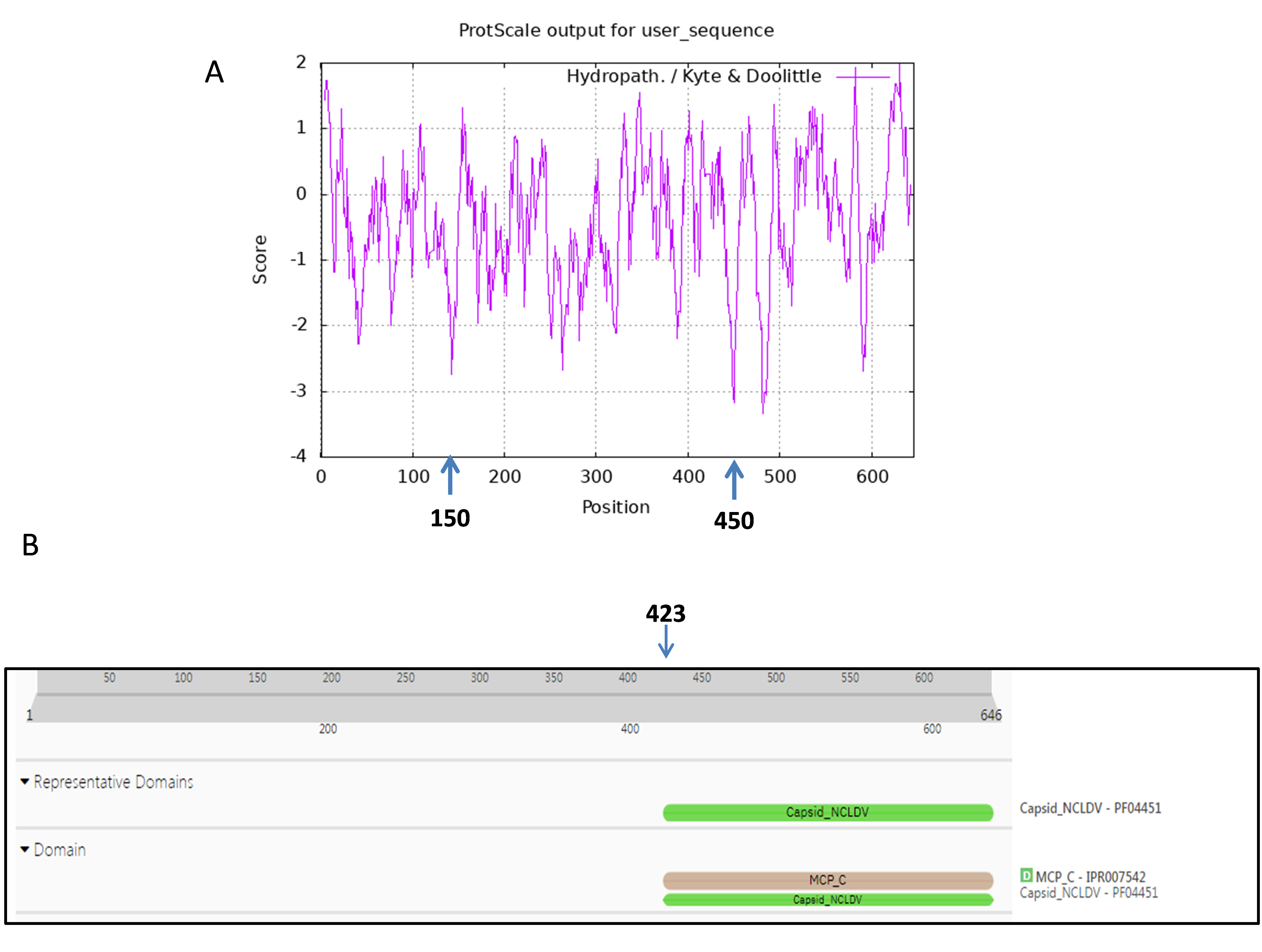

Supplement: Supplementary file 7 — Additional file 7. Hydrophilicity, hydrophobicity and functional domain analysis of p72. A The hydrophilicity and hydrophobicity of p72 were analysed with the ExPASy online tool to determine the potential highly expressed domain. B The functional domain of p72 was predicted by the InterPro online tool, and functional domain prediction was based on its sequence similarity with other NCLDV family members. The boundaries of the truncations are indicated by the arrows. [file 13567_2024_1407_MOESM7_ESM.tif]

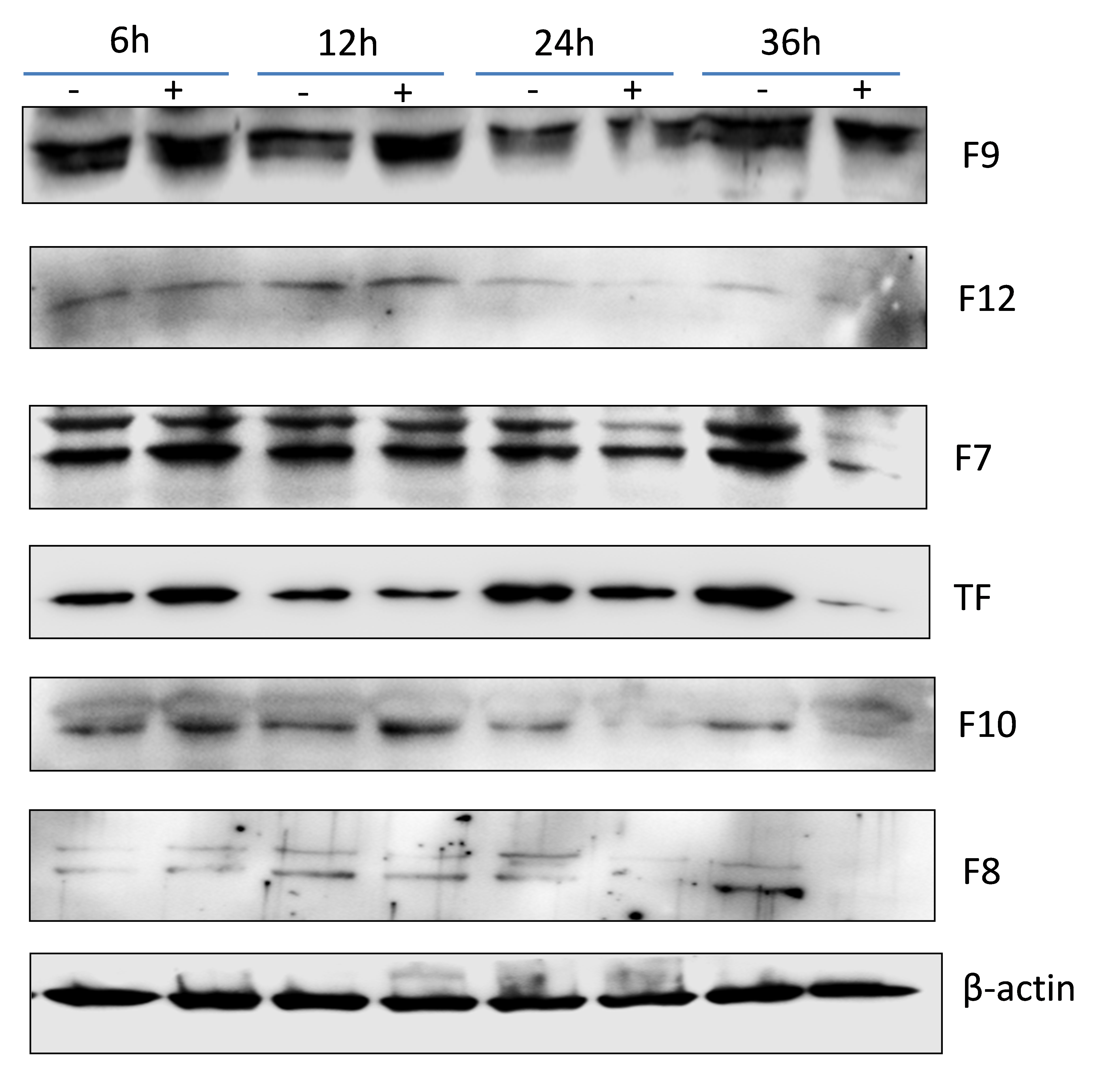

Supplement: Supplementary file 8 — Additional file 8. ASFV recombinant strain infection regulated coagulation factor expression. PAM cells were infected with 0.1 MOI ASFV for 6, 12, 24 or 36 h. Intrinsic coagulation factors, crucial coagulation factor F10 and extrinsic coagulation factors were detected by western blotting at the indicated time points. [file 13567_2024_1407_MOESM8_ESM.tif]
